# EEG Lempel-Ziv complexity varies with sleep stage, but does not seem to track dream experience

**DOI:** 10.3389/fnhum.2022.987714

**Published:** 2023-01-10

**Authors:** Arnfinn Aamodt, André Sevenius Nilsen, Rune Markhus, Anikó Kusztor, Fatemeh HasanzadehMoghadam, Nils Kauppi, Benjamin Thürer, Johan Frederik Storm, Bjørn Erik Juel

**Affiliations:** ^1^Brain Signalling Lab, Division of Physiology, Faculty of Medicine, Institute of Basic Medical Sciences, University of Oslo, Oslo, Norway; ^2^National Centre for Epilepsy, Oslo University Hospital, Oslo, Norway; ^3^School of Psychological Sciences, Monash University, Clayton, VIC, Australia

**Keywords:** consciousness, sleep, dreaming, EEG, complexity

## Abstract

In a recent electroencephalography (EEG) sleep study inspired by complexity theories of consciousness, we found that multi-channel signal diversity progressively decreased from wakefulness to slow wave sleep, but failed to find any significant difference between dreaming and non-dreaming awakenings within the same sleep stage (NREM2). However, we did find that multi-channel Lempel-Ziv complexity (LZC) measured over the posterior cortex increased with more perceptual ratings of NREM2 dream experience along a thought-perceptual axis. In this follow-up study, we re-tested our previous findings, using a slightly different approach. Partial sleep-deprivation was followed by evening sleep experiments, with repeated awakenings and immediate dream reports. Participants reported whether they had been dreaming, and were asked to rate how diverse, vivid, perceptual, and thought-like the contents of their dreams were. High density (64 channel) EEG was recorded throughout the experiment, and mean single-channel LZC was calculated for each 30 s sleep epoch. LZC progressively decreased with depth of non-REM sleep. Surprisingly, estimated marginal mean LZC was slightly higher for NREM1 than for wakefulness, but the difference did not remain significant after adjusting for multiple comparisons. We found no significant difference in LZC between dream and non-dream awakenings, nor any significant relationship between LZC and subjective ratings of dream experience, within the same sleep stage (NREM2). The failure to reproduce our own previous finding of a positive correlation between posterior LZC and more perceptual dream experiences, or to find any other correlation between brain signal complexity and subjective experience within NREM2 sleep, raises the question of whether EEG LZC is really a reliable correlate of richness of experience as such, within the same sleep stage.

## 1. Introduction

According to minimal forms of physicalism, there can be no change in the mental properties of the world, without some change in the physical properties of the world^1^. More narrowly, assuming that your brain encompasses the part of the physical world directly^2^ relevant to your consciousness, it follows that the vast range of distinct experiences available to you must supervene on a similarly rich repertoire of possible configurations of your brain.^3^

While conscious experience may be incredibly diverse, each experience is nevertheless perceived as a unified whole. If we commit to stronger physicalist assumptions linking properties of phenomenology to properties of the physical world, it seems plausible that our integrated subjective experience must be supported by physical integration of information in the brain.

Perhaps inspired by intuitions like these, many researchers and theories of consciousness have (to varying degrees) emphasized phenomenological integration and/or differentiation as important features of our experience that should be accounted for by scientific theories of consciousness,^4^ and have attempted to formalize these intuitions in terms of information, entropy, complexity, and causality (Baars, 1988; Chalmers, 1995; Tononi and Edelman, 1998; Dehaene and Naccache, 2001; Edelman, 2003; Seth and Baars, 2005; Tononi, 2005; Seth et al., 2006, 2008; Barrett, 2014; Carhart-Harris et al., 2014; Ruffini, 2017; Sarasso et al., 2021).

More specifically, there seem to be a convergence of opinions and evidence toward the hypothesis that conscious experience is supported by complex dynamics of the thalamo-cortical system, a part of the brain argued to be particularly suited to integrate information (across multiple cortical areas and modalities) (Crick and Koch, 1990; Llinás et al., 1998; Mashour, 2004; Baars, 2005; Seth et al., 2005, 2006; Tononi, 2005; Baars et al., 2013; Sarasso et al., 2021) [but see Merker (2007)].

Observations supporting this hypothesis goes back to the very beginnings of human electroencephalography (EEG) (Gibbs et al., 1935), as reflected by the classical distinction between the “activated” (Moruzzi and Magoun, 1949) chaotic, low-amplitude, fast EEG of wakefulness and the regular, high-amplitude, slow EEG of deep sleep, anesthesia, and coma (Seth and Baars, 2005).

More recently, complexity theories of consciousness, such as the Integrated Information Theory (IIT) (Tononi, 2004; Oizumi et al., 2014) and the Entropic Brain Hypothesis (EBH) (Carhart-Harris et al., 2014; Carhart-Harris, 2018), have received considerable support from systematic between-states comparisons. For example, the Perturbational Complexity Index (PCI), inspired by IIT, has shown an impressive ability to distinguish and stratify different brain states such as wakefulness, REM-sleep, deep sleep, anesthesia, and different disorders of consciousness (DOCs) according to the “level” of consciousness ascribed to each of these states (Casali et al., 2013; Casarotto et al., 2016). By measuring the LZC of the average cortical response to repeated electromagnetic stimulation, PCI aims to average out ongoing activity and capture the complexity of the “deterministic” effects of the perturbation, thus quantifying the capacity of the system for both integration and differentiation.

While LZC and other measures of signal diversity applied to spontaneous brain activity do not capture (the capacity of the system for) causal integration in the same way,^5^ they are easier to use, and have also been found to be lower in deep sleep and anesthesia compared to wakefulness and REM sleep, and elevated in psychedelic states, associated with reports of an increased range or richness of conscious contents (Schartner, 2017; Timmermann et al., 2019). Importantly, measures of spontaneous signal diversity seem more likely than PCI to reflect ongoing experience. Indeed, the LZC of EEG and magnetoencephalography (MEG) recordings has been found to correlate with self-reported intensity of drug-induced psychedelic experience (Schartner et al., 2017a; Timmermann et al., 2019).

However, there are a lots of differences between distinct global physiological states, many of which may have little or no direct relation to conscious experience. Ideally, we would like measures of consciousness to capture changes in experience within (approximately^6^) the same global state. It is not yet clear whether signal diversity measures are able to do this. For example, while Timmermann et al. (2019) observed that the variation in LZC over time mirrored the self-reported intensity of the ongoing psychedelic experience, Bola et al. (2018) found LZC to be unaffected by the presentation rate of audio stimuli (recordings of book excerpts).

Inspired by these developments, and a promising serial awakening paradigm (Siclari et al., 2013, 2017), we recently conducted a small study of EEG signal diversity [multi-channel Lempel Ziv complexity, amplitude coalition entropy and synchrony coalition entropy (Schartner, 2017)] in sleep and dreaming (Aamodt et al., 2021). Participants slept in the lab in the morning after a full night of sleep deprivation, while we recorded their EEG, and intermittently woke them up for an immediate dream report. We found that signal diversity decreased with increasing depth of non-REM sleep, but failed to find any significant difference between dreaming and non-dreaming within the same state (stage NREM2 sleep). However, we did find that multi-channel LZC measured over the posterior cortex increased with more perceptual (as opposed to thought-like) ratings of dream experience.

In this follow up study, we wanted to re-test our previous findings using a slightly different paradigm. In order to adhere more closely to the natural circadian rhythm, full sleep deprivation and morning sleep was replaced by partial sleep deprivation and evening sleep. The dream questionnaire was updated to ask for explicit ratings of dream experience diversity and vividness, and the rating of dream experience along a single axis as more thought-like versus more perceptual was replaced by two separate ratings of how perceptual and how thought-like the dream experience was. Instead of the three multichannel diversity measures used in the first study we chose to focus on single-channel LZC (based on promising results from earlier research (Schartner et al., 2017a; Timmermann et al., 2019) and suggestions from reviewers of our first sleep study), and take a simple average over all channels included in each channel selection.

Starting from the idea that–under certain conditions–complexity of brain activity may correlate with richness of our subjective experience, and the conjecture that the complexity of brain activity may correlate with the LZC of EEG signals,^7^ we derived and tested four hypotheses about how EEG complexity varies with sleep stages and dream experience (within the same stage):

1. Mean EEG complexity over all channels should be higher for wakefulness (and REM sleep), and progressively decrease with increasing depth of non-REM sleep.
2. Within the same sleep stage, EEG complexity measured over the posterior hot zone (PHZ), suggested to most directly support conscious experience (Koch et al., 2016; Boly et al., 2017; Siclari et al., 2017; Storm et al., 2017), should:
  a. Be higher for awakenings following an epoch of dreaming, versus awakenings following an epoch without dreaming.
  b. Increase with richness of dream experience, as measured by subjective ratings of diversity and vividness of dream contents.
  c. Increase with ratings of dream experience as more perceptual.

In addition to testing these four hypotheses, we checked how the results for mean single-channel LZC compared to those for the multi-channel LZC measure used in our previous study (Aamodt et al., 2021), and we explored how variation in LZC compared to changes in the slope of the frequency spectrum, quantified by the aperiodic spectral exponent [which has itself been suggested as a measure of consciousness (Colombo et al., 2019)]. We analyzed topographical patterns in EEG complexity, related to sleep and dreaming, and briefly explored how ratings of dream content vary with sleep stages.

## 2. Materials and methods

The experimental paradigm and the overall methodological approach were largely similar to our previous study of morning sleep in fully sleep-deprived participants (Aamodt et al., 2021), except for the differences briefly summarized in the Section “1 Introduction.” For readability and completeness, this section includes some repetitions of the description in Aamodt et al. (2021).

### 2.1. Experimental paradigm

Inspired by Siclari et al. (2013), we employed an evening sleep paradigm with remotely monitored sleep EEG and repeated awakenings, each followed by an immediate dream report.

Before the day of the sleep experiment, we met with the participants, explained the experiment, and showed them the sleep lab.

Participants were told to go to bed at their usual bedtime, and to get up 3 h earlier than their usual rising time, before meeting in the lab (partially sleep-deprived) at 19:30 in the evening. They were instructed to avoid alcohol (and drugs) the last 48 h before the experiment, and caffeine the last 12 h before the experiment. Prior to the experiment, the questionnaire used for the dream reports was emailed to all participants, together with detailed explanations of how to interpret each question. Participants were reminded to read the descriptions and instructions, and to practice answering the questions.

After meeting in the lab on the day of the experiment, the EEG cap was mounted on the participant’s head while we went through the dream questionnaire, cleared up any lingering uncertainties and did a practice run through the questions. We also reassured the participants that we had already accounted for the fact that some participants will not be able to sleep while planning the experiment, and that there were no wrong answers to the questions, as long as they were honest. Toward the end of the preparation, we made sure to start dimming the lights and instructed the participants to try to go to the bathroom.

The researchers then left the room, and lights were turned off. Via a remote audio connection, participants were first told to lie still with eyes open (EO) for 5 min of awake EEG recording. Second, they were told to repeat the exercise with eyes closed (EC), for another 5 min of awake EEG recording, interrupted by an electronic alarm and subsequent awake (baseline) experience report using the dream questionnaire.^8^ Finally, they were told that they were free to fall asleep.

While the participants slept, EEG was remotely monitored from another room. Participants were intermittently awakened, followed by immediate dream experience reports. In order to get many awakenings within the same sleep stage and increase the statistical power of the within-state analysis, most awakenings were performed during NREM2 sleep.^9^ In our previous morning sleep study, we focused on NREM2 sleep in an attempt to optimize the balance of dream and non-dream awakenings. While we in fact had very few non-dream awakenings in our first study, other sleep stages were not much better, and our experience was that many participants were able to reach the (intermediate depth) sleep stage NREM2 fairly quickly, wake up easily enough and give clear reports, and fall back asleep without too much trouble. Hence, we chose to keep the focus on NREM2 sleep.

### 2.2. Participants and data

**FIGURE 1:**
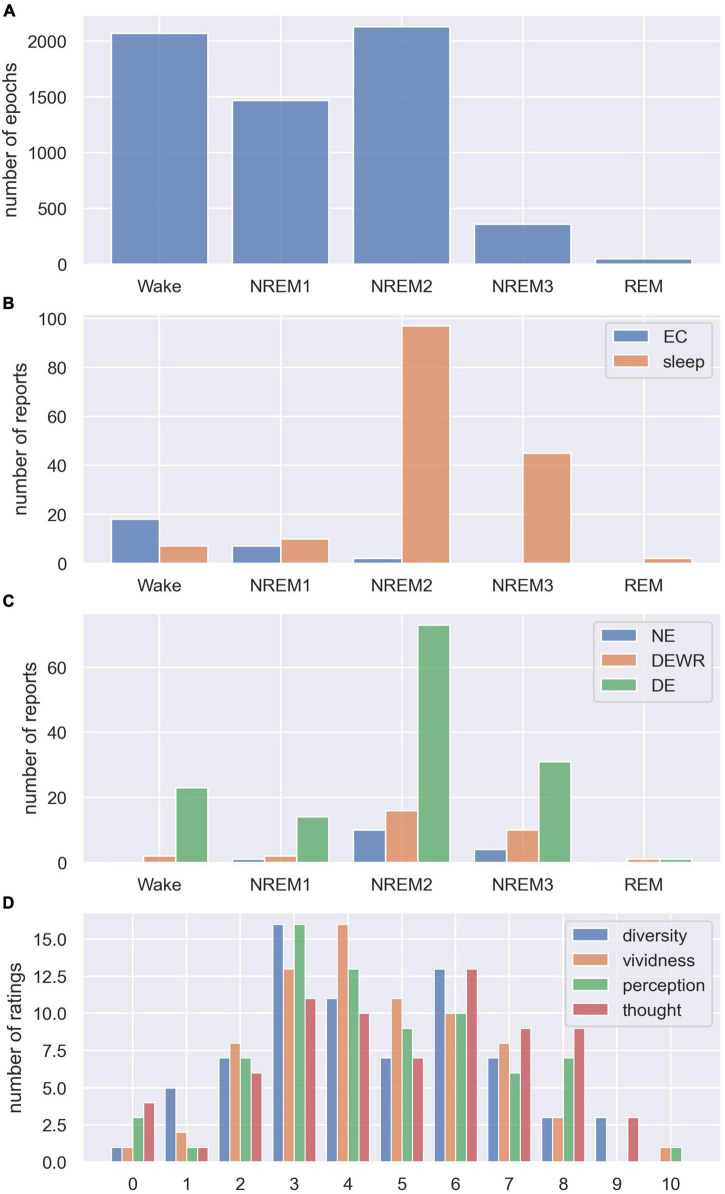
Distribution of sleep stages, dream experience classifications, and subjective ratings. **(A)** Total number of scored 30 s sleep epochs from each sleep stage (summed over all participants and trials). **(B)** Number of subjective reports collected from each sleep stage (stage of last epoch before alarm) for eyes closed (EC) and sleep trials (reports were not collected from EO trials). **(C)** Number of subjective reports from each sleep stage classified as no experience (NE), dream experience without recall of contents (DEWR) and dream experience with recall of contents (DE). **(D)** Distribution of subjective ratings from NREM2 DE awakenings, showing how diverse, vivid, perceptual, and thought-like NREM2 dream contents were, on a scale from 0 to 10 (see section “2.4. Dream report questionnaire” for detailed explanation of the scale).

We recruited 27 participants for the sleep experiment. Five minutes of EO/EC resting EEG trials were recorded before the start of the sleep experiment (28 EO trials, 28 EC trials).^10^ We recorded 164 sleep trials using the intermittent awakening paradigm described above, where sleep trial refers to the period from the participant was told to go to sleep until they were awakened by the alarm.^11^ From all the recordings, we obtained a total of 6,071 scored 30-s sleep epochs (Wake = 2,070, NREM1 = 1,469, NREM2 = 2,130, NREM3 = 356, REM = 46, see Figure 1A), used for the analysis of how LZC varied with sleep stage. There were 27 subjective reports from the eyes closed trials at the start of the experiment (Wake = 18, NREM1 = 7, NREM2 = 2, see Figure 1B), and 161 reports from the subsequent sleep trials (Wake = 7, NREM1 = 10, NREM2 = 97, NREM3 = 45, REM = 2, see Figure 1B).^12^ For the analysis of whether LZC, within the same sleep stage, was different between awakenings with no reported experience (NE), dream experience without recall (DEWR), and dream experience (DE) with explicit recall of contents, we used the 99 awakenings from NREM2 sleep (NE = 10, DEWR = 16, DE = 73, see Figure 1C). Finally, for the 73 NREM2 DE awakenings, we analyzed the relationship between LZC and subjective ratings of dream experience diversity and vividness, as well as ratings of how perceptual the dream was (see Figure 1D for distribution of NREM2 dream content scores).

### 2.3. Recording and processing of EEG

A total of 62 EEG channels and two electrooculogram (EOG) channels, referenced to an electrode placed medially on the forehead, were recorded at a 1,000 Hz sampling rate using two 32 channel amplifiers (BrainAmp DC, Brain Products GmbH, Gilching, Germany). EOG channels were placed in the American Academy of Sleep Medicine (AASM) recommended E_1_ and E_2_ positions below and above the outer canthi (Berri et al., 2018). Two separate bipolar electrodes on either side of the mentalis muscle on the front of the chin were used to record the electromyogram (EMG).

The time of the electronic alarm was recorded during the experiment by manually pressing a button to insert a marker into the EEG recording right before triggering the alarm, and an adjusted (if necessary) time of awakening was determined by offline inspection of the EEG recording. The beginning of the recording was then cropped to get a whole number of sleep epochs, here used to denote the 30 s segments of EEG conventionally used for scoring of sleep stages.

For the purpose of sleep scoring, we exported a separate copy of the recording with the recommended (F4-M1, C4-M1, O2-M1) and backup (F3-M2, C3-M2, O1-M2) AASM bipolar leads. Sleep scoring was performed (by RM) according to “The AASM Manual for Scoring of Sleep and Related Events version 2.5” (Berri et al., 2018), and the hypnograms were then reviewed by another researcher (AAa).

After cropping the original EEG recording to a whole number of sleep epochs before time of awakening, bad segments of the data (e.g., containing large muscle artifacts, movement, etc.) were identified by manual inspection and marked. Some recordings contained large jumps due to DC correction performed mid-recording. These jumps were interpolated to avoid large ringing artifacts from subsequent temporal filters, and the segment of EEG containing the jump was excluded from subsequent analysis. Data was then exported for automatic pre-processing using the EEGLAB MATLAB toolbox (Delorme and Makeig, 2004).

First, data was down-sampled to 250 Hz using a mild anti-aliasing filter. Bad channels were automatically detected and interpolated, and robust average re-referencing was performed using the EEG-Clean-Tools EEGLAB-plugin (PREP-pipeline) (Bigdely-Shamlo et al., 2015).

Afterward, bad segments were (temporarily) removed from the recording, and data was high-pass filtered at 0.75 Hz, before running independent component analysis (ICA) using the extended infomax algorithm (Bell and Sejnowski, 1995). The ICLabel EEGLAB plugin (Pion-Tonachini et al., 2019) was used to automatically assign probable sources (brain, muscle, eye, heart, line nose, channel noise, or other) to each independent component. Independent components were then rejected based on their ICLabel-assigned probabilities if they met any of the following three criteria:

1. The most likely heart component of the recording and assigned probability of heart being the source of the component higher than 10%,
2. Less than 15% assigned probability of brain being the source of the component, or
3. Combined assigned probability of stemming from non-brain sources was more than half the assigned probability that the component came from brain sources.

After ICA-cleaning, bad segments were re-inserted into the recording (this was only done to keep original length of recording and timing of events, as calculation windows intersecting bad segments were later dropped), and data was low-pass filtered at 40 Hz.

Electroencephalography recordings were re-imported into MNE Python, and cleaning performance was manually inspected. A few recordings still had very pronounced heart artifacts, probably because the heart artifact had been split into several independent components during ICA-cleaning, and only one of these had been rejected by the heuristic rules above. For these recordings, IC-components were manually re-inspected and remaining heart components were rejected. Finally, any remaining bad segments of the recordings were marked for rejection, and remaining bad channels were interpolated.

### 2.4. Dream report questionnaire

Immediately after each awakening, participants were asked to report and rate any dream experience they might have had. To keep awakenings brief, we used only the abbreviated short form prompts shown in parentheses in the dream questionnaire below (see Supplementary materials for the full text sent to participants before the experiment):

1. What was the last thing going through your mind? (Last experience?)

Describe the most recent experience (for example image, thought or emotion) which you had before the alarm sound (but after falling asleep), if you experienced anything at all–it is just as fine if you did not experience anything. By experience we mean “any kind of mental activity,” including thoughts, dreams, perceptions, emotions, etc. (e.g., if asked while being awake, the last thing going through your mind might just be the scene you see in front of you).

2. What was the last emotion you had? (Last emotion?)

Describe your most recent feelings or emotions (for example anger, disgust, fear, happiness, sadness, surprise etc.) which you had before the alarm sound (but after falling asleep), if you experienced any emotions at all–it is just as fine if you did not experience any emotions.

3. How diverse was your experience on a scale from 0 to 10? (Diversity 0–10?)

Please rate your experience from 0 to 10, where 0 means “I did not experience anything at all” and 10 means “my experience was just as diverse or more diverse than my normal waking experience.” The more different emotions, sensory impressions, objects, actions, thoughts, your experience consists of, the more diverse (rich, varied) we consider the experience to be.

4. How vivid was your experience on a scale from 0 to 10? (Vividness 0–10?)

Please give a rating of your experience from 0 to 10, where 0 means “I did not experience anything at all” and 10 means “my experience was just as vivid or more vivid than my normal waking experience.” The clearer and more life-like your experience, the more vivid (as opposed to blurry and diffuse) we consider the experience to be.

5. How perceptual was your experience on a scale from 0 to 10? (Perceptual 0–10?)

Please rate the extent to which your experience was perceptual in character. In this context, perceptual refers to any visual, auditory, olfactory, or bodily experience/imagery that you had.

6. How thought-like was your experience on a scale from 0 to 10? (Thought-like 0–10?)

Please rate how thought-like your experience was. Thought-like refers to reasoning or other mental processes not necessarily related to/accompanied by perceptual experiences/imagery (or inner speech).

Based on the answer to Question 1, awakening reports were classified as indicating no experience (NE), dream experience without recall (DEWR), or dream experience (DE). Subjective reports that included explicit recall of any dream contents at all, were scored as DE. If the participant reported having had a dream, but were unable to remember any specific contents, the report was scored as DEWR. If necessary, participants were asked to disambiguate between NE and DEWR, before continuing the awakening interview.

While interesting, we chose to exclude Question 2 about the last emotion before awakening from further analysis, because we were unsure whether all (or even most) of these answers referred to the period right before the awakening, rather than the time of questioning (a large portion of the answers mentioned feeling tired and annoyed). Question 6 was only included in the exploratory analysis, since we did not feel that a clear hypothesis could be derived about how thought-like dream experiences should relate to EEG complexity.

### 2.5. Lempel-Ziv complexity

The EEG recording was divided into 8 s windows, with 7 s overlap, for calculation of single-channel LZC. Any window containing stretches of the recording marked as bad was excluded from further analysis. For each window and each channel, LZC calculation was based on the complex-valued analytical representation of the signal. The analytical representation of the signal has the original EEG signal as its real component, and the Hilbert transformed signal as its imaginary component. This analytical representation of the EEG signal can be expressed as the (pointwise) product of a time varying real-valued, positive amplitude, and a time varying complex-valued phase angle. The analytical signal was binarized by thresholding on the median value of its amplitude (within the window), and the complexity or compressibility of the resulting binary string was estimated by the LZC algorithm.

Roughly speaking, the LZC algorithm works by splitting a binary string into distinct substrings that together allows reconstruction of the whole string. Starting from the beginning of the binary string, the algorithm iteratively builds a dictionary representation of the string in terms of unique substrings. The length of this dictionary serves as a measure of how complex or incompressible the string is. Here, we normalized the length of the dictionary produced by LZC, by the length of the dictionary produced by LZC on a randomly shuffled version of the original string, yielding a measure that is (almost always) between 0 and 1. Intuitively, a simple string with stereotyped patterns will allow a very simple representation, while completely random strings will be (among) the most difficult ones to represent. See for example (Lempel and Ziv, 1976; Schartner, 2017) for further details, and Figure 2 in Schwartzman et al. (2019) for a schematic representation of how the LZC value of the EEG signal is calculated.^13^

### 2.6. Aperiodic spectral exponent

For each 8 s window and each channel, we also calculated the exponent of the aperiodic component of the EEG power spectrum. The FOOOF algorithm (version 1.0.0) was used to parameterize EEG power spectra by a model composed of an aperiodic power law component, together with a periodic component consisting of zero or more oscillatory Gaussian peaks (Donoghue et al., 2020). We limited peak width to 3–8 Hz, and we set a relative threshold for peak detection to three standard deviations of the input data. Power spectra were parameterized across the frequency range 0.75–40 Hz.

While we calculated the exponent of the aperiodic component of the frequency spectrum because we wanted to get a sense of the extent to which LZC offers advantages beyond measures based on gross characteristics of the power spectrum,^14^ the spectral exponent has also been suggested as a measure of consciousness in its own right (Colombo et al., 2019).

### 2.7. Statistical analysis

Statistical analysis was performed using IBM SPSS version 28. The relationship between sleep stage and whole-brain LZC was assessed using a linear mixed model (LMM)^15^ with sleep stage as a fixed factor (including intercept), and participant and trial (nested within participants) as random intercepts. Sleep epochs were entered as repeated measures with first-order auto regressive residual variance-covariance.

For awakenings in NREM2 sleep, the relationship between experience report (NE, DEWR, or DE) and posterior LZC of the last sleep epoch before awakening was assessed using a LMM with experience classification as the fixed factor (including intercept) and subject as random intercept.

For NREM2 awakenings with reported dream experience (DE), the correlation between subjective ratings of richness of experience and posterior LZC values was assessed using a LMM with the subjective ratings of diversity and vividness as fixed continuous covariates (including intercept, but without interaction term) and subject as random intercept.

For NREM2 awakenings with reported dream experience (DE), the correlation between subjective ratings of how perceptual experience was and posterior LZC values were assessed using a LMM with the subjective rating as a fixed continuous covariate (including intercept) and subject as random intercept.

We used the inheritance procedure (Goeman and Finos, 2012) to adjust for multiple comparisons and control the family-wise error rate. Starting with significance level α = 0.05, this yielded an adjusted significance level α/4 = 0.05/4 = 0.0125 for models of whole-brain/posterior LZC as functions of sleep stage, experience classification, subjective rating of experiential richness and subjective ratings of how perceptual the experience was and α/(4*10) = 0.05/(4*10) = 0.00125 for each of the 10 pairwise comparisons between different sleep stages (adjustments for other more detailed tests ended up being irrelevant).

## 3. Results

### 3.1. Hypothesis testing

#### 3.1.1. Does LZC decrease with depth of NREM sleep?

**FIGURE 2:**
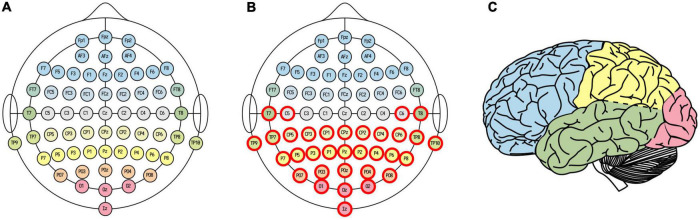
EEG channel selections. Whole-brain **(A)** and posterior **(B)** channel selections. Electrode fill color indicates associated cortical lobe **(C)** [adapted from illustration by Laurens R. Krol, distributed under a CC0 1.0 license (Krol, 2020)].

**FIGURE 3:**
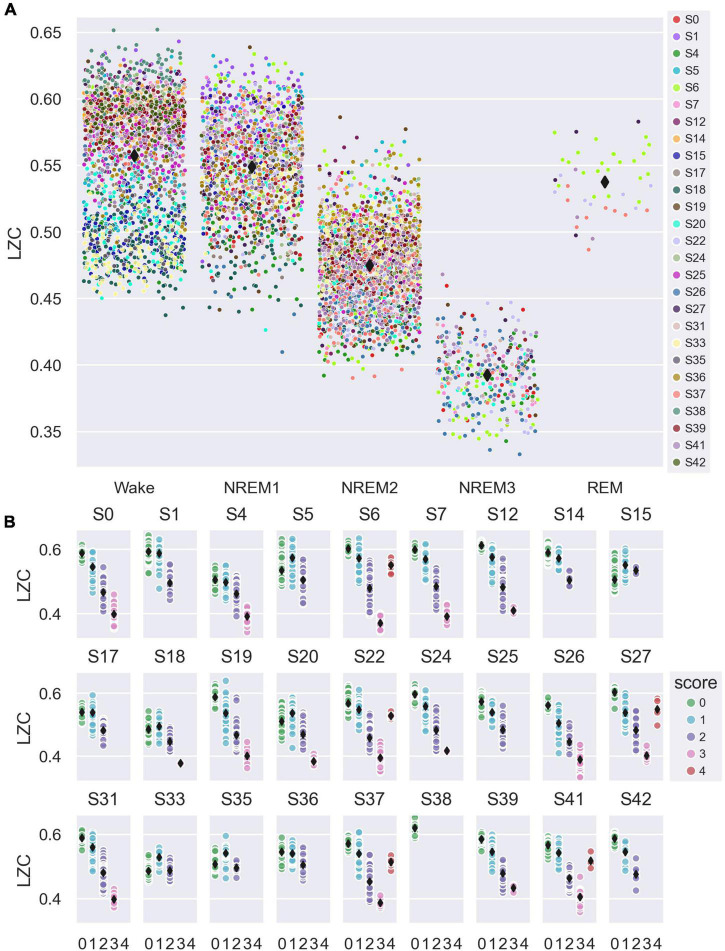
Variation in whole-brain average single-channel Lempel-Ziv complexity (LZC) with sleep stage. **(A)** Average single-channel LZC vs. sleep stage for the whole-brain channel selection (all channels, see Figure 2A). Each data point corresponds to one 30 s sleep epoch. Observations are randomly scattered along the *x*-axis to reduce overlap, and participant number is indicated by marker fill color. Overall mean values for each sleep stage are indicated by black diamond markers. **(B)** Whole-brain average single-channel LZC vs. sleep stage (0 = W, 1 = NREM1, 2 = NREM2, 3 = NREM3, 4 = REM), plotted separately for each study participant. Each data point corresponds to one 30 s sleep epoch. Fill color indicates sleep stage, and black diamond markers indicate participant mean values for each stage.

For the analysis of how LZC varied with sleep stage, we averaged single-channel LZC over all available channels (whole-brain channel selection in Figure 2A), and over all 8 s sliding calculation windows (7 s overlap) fully contained within each 30 s sleep epoch, to yield a single LZC value per sleep epoch. We included all available scored sleep epochs in the analysis, from both EO/EC and sleep trials. Figure 3 shows how whole-brain mean LZC values were distributed across sleep stages, plotted for all participants together (A) and for each participant separately (B). Table 1 summarizes the results of the statistical analysis.

**TABLE 1:** Whole-brain average single-channel Lempel-Ziv complexity (LZC) as function of sleep stage.

Response	Fixed effect	df1	df2	*F*	Sig.					
LZC	Intercept	1	27	8,285	<0.0001					
	Sleep stage	4	3,809	490	<0.0001		**Sig. (pairwise comparison)**			
	**EMMs**	**Estimate**	**Std. err.**	**CI_lower_**	**CI_upper_**		**NREM1**	**NREM2**	**NREM3**	**REM**
	Wake	0.541	0.006	0.529	0.552		0.008	<0.001	<0.001	0.214
	NREM1	0.544	0.006	0.532	0.555			<0.001	<0.001	0.081
	NREM2	0.496	0.006	0.485	0.508				<0.001	<0.001
	NREM3	0.451	0.006	0.439	0.463					<0.001
	REM	0.532	0.008	0.516	0.549					
	**Random effect covariance**		**Estimate**	**Std. err.**	***Z***	**Sig.**	**CI_lower_**	**CI_upper_**		
	Variance (participant)		7.23e-4	2.33e-4	3.10	0.002	3.85e-4	0.0014		
	Variance (participant * trial)		4.85e-4	7.48e-5	6.49	<0.001	3.59e-4	6.56e-4		

Summary of results from the linear mixed model (LMM) of whole-brain average single-channel LZC as a function of sleep stage (see section “2. Materials and methods” for further details).

Sleep stage was a significant factor in the model for between-states variation in whole-brain mean LZC [*F*_(4,3809)_ = 490, *p* < 0.001]. The estimated marginal mean LZC for NREM3 was lower than for wake, and the difference (Δ = 0.090) was about three times the estimated between-participant standard deviation (*s* = 0.0269), and four times the estimated between-trials standard deviation (*s* = 0.0220). Estimated marginal mean LZC was progressively lower for increasing depth of NREM sleep, but were (slightly) higher for NREM1 than wake and REM, in contrast to expectations (raw means were still higher for Wake than for NREM1 for most participants). All pairwise contrasts between different sleep stages were significant, except the REM-wake and REM-NREM1 contrasts. However, the difference between NREM1 and wakefulness was no longer significant after multiple comparison correction (adjusted significance level α/(4*10) = 0.00125, see Section “2 Materials and methods” for details).

#### 3.1.2. Is posterior LZC higher for dreaming than non-dreaming?

**FIGURE 4:**
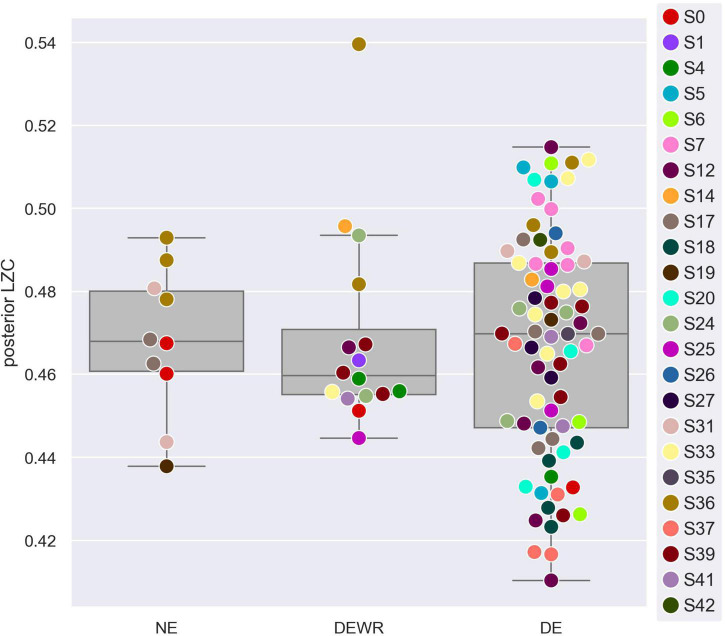
Posterior average single-channel Lempel-Ziv complexity (LZC) versus NREM2 dream experience classification. Average single-channel LZC vs. NREM2 dream experience class (NE, no experience; DEWR, dream experience without recall of contents; DE, dream experience), for the posterior EEG channel selection (see Figure 2B). Each data point corresponds to the last 30 s sleep epoch before an awakening from NREM2 sleep. Observations are plotted on top of corresponding boxplots. Participant number is indicated by marker fill color, and observations are displaced slightly along *x*-axis to avoid overlap.

For the analysis of how LZC of the last epoch before awakening from NREM2 sleep varied with dream experience classification and dream content ratings, we used a posterior channel selection (Figure 2B) covering most of the temporo-parietal-occipital lobes (see Figure 2C). There were 99 awakening reports immediately following an epoch of NREM2 sleep. Figure 4 shows the distribution of posterior LZC values for each dream experience class, and Table 2 summarizes the results of the statistical analysis. There was no significant (or near significant) difference in posterior mean LZC between NE and DE awakenings from NREM2 sleep.

**TABLE 2:** Posterior average single-channel Lempel-Ziv complexity (LZC) as a function of NREM2 dream experience class.

Response	Fixed effect	df1	df2	*F*	Sig.				
LZC_posterior_	Intercept	1	40.6	11,251	<0.0001				
	Experience class	2	93.4	0.233	0.793		**Sig. (pairwise comparison)**		
	**EMMs**	**Estimate**	**Std. err.**	**CI_lower_**	**CI_upper_**			**DEWR**	**DE**
	NE	0.461	0.008	0.445	0.478			0.527	0.523
	DEWR	0.468	0.007	0.455	0.481				0.900
	DE	0.467	0.004	0.459	0.475				
	**Random effect covariance**		**Estimate**	**Std. err.**	***Z***	**Sig.**	**CI_lower_**	**CI_upper_**	
	Variance (participant)		1.73e-4	8.68e-5	1.99	0.046	6.46e-5	4.62e-4	

Summary of results from the linear mixed model (LLM) of posterior average single-channel LZC as a function of dream experience class (see section “2. Materials and methods” for further details), for awakenings from NREM2 sleep. The data for the DEWR category contained an outlier (see Figure 4). Excluding this data point gave estimated marginal mean posterior LZC for DEWR between the values for NE and DE, but results were otherwise similar.

#### 3.1.3. Does posterior LZC correlate with dream content ratings?

**FIGURE 5:**
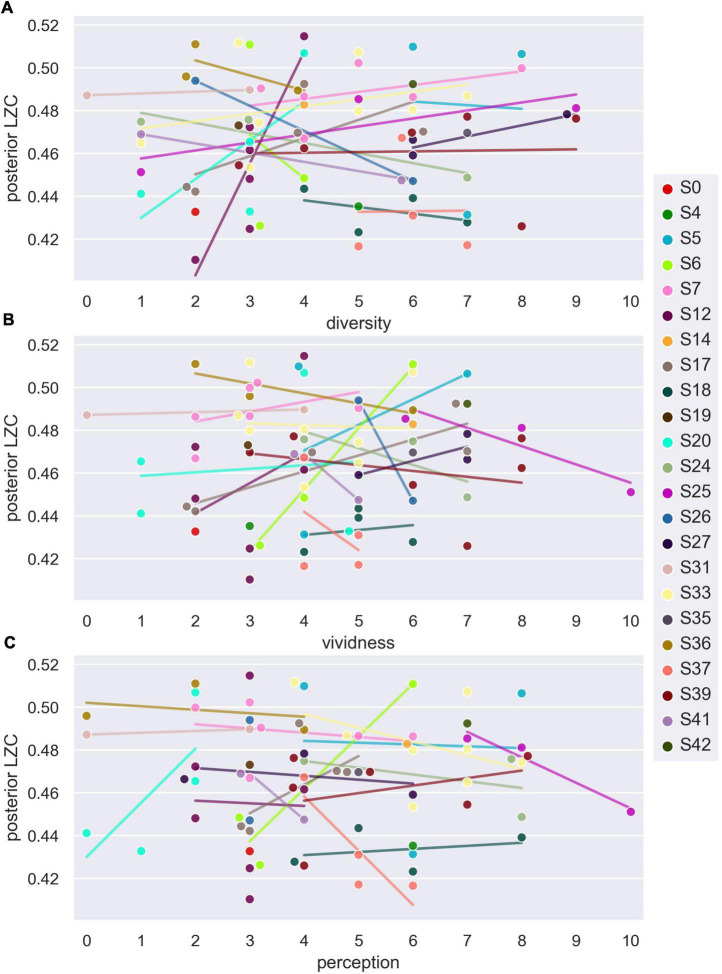
Average posterior single-channel Lempel-Ziv complexity (LZC) versus subjective ratings of NREM2 dream experience. Posterior LZC vs. ratings of how diverse **(A)**, vivid **(B)**, and perceptual **(C)** NREM2 dream experience was. Each data point corresponds to the last 30 s sleep epoch before an awakening from NREM2 sleep. Participant number is indicated by marker fill color, and observations are displaced slightly along *x*-axis to avoid overlap. Linear trend for each participant (for which there is more than one data point) is indicated by background line segments (visual aid only).

For the analysis of how posterior LZC varies with subjective ratings of dream content, we used the 73 DE awakenings from NREM2 sleep. Figure 5 shows the variation in posterior LZC with subjective ratings of how diverse, vivid, and perceptual the dream experience was. Table 3 summarizes the results of the two statistical models of variation in posterior LZC with dream experience richness (diversity and vividness) and ratings of how perceptual the experience was. There was no significant (or near significant) relationship between posterior mean LZC and subjective ratings of NREM2 dream experience.^16^

**TABLE 3:** Posterior average single-channel Lempel-Ziv complexity (LZC) as a function of subjective ratings of NREM2 dream contents.

Response	Fixed effect	df1	df2	*F*	Sig.	Estimate	Std. err.	*t*	CI_lower_	CI_upper_
LZC_posterior_	Diversity	1	68.9	0.044	0.835	0.0033	0.0160	0.209	−0.0286	0.0352
	Vividness	1	70.0	0.527	0.470	0.0133	0.0183	0.726	−0.0232	0.0497
	(Intercept)	1	57.9	2,243	<0.0001	0.4592	0.0097	47.4	0.4398	0.4787
	**Random effect covariance**		**Estimate**	**Std. err.**	***Z***	**Sig.**	**CI_lower_**	**CI_upper_**		
	Variance (participant)		0.0002	0.0001	1.58	0.114	5.21e-5	0.0006		
**Response**	**Fixed effect**	**df1**	**df2**	***F***	**Sig.**	**Estimate**	**Std. err.**	***t***	**CI_lower_**	**CI_upper_**
LZC_posterior_	Perception	1	46.8	0.007	0.936	−0.0013	0.0162	−0.081	−0.0337	0.0311
	(Intercept)	1	58.1	3,176	<0.0001	0.4673	0.0083	56.4	0.4506	0.4840
	**Random effect covariance**		**Estimate**	**Std. err.**	***Z***	**Sig.**	**CI_lower_**	**CI_upper_**		
	Variance (participant)		0.0002	0.0001	1.53	0.127	4.56e-5	0.0006		

Summary of results from a linear mixed model (LLM) of posterior average single-channel LZC as a function of subjective ratings of dream experience diversity and vividness, and a similar model of posterior LZC as a function of ratings of how perceptual dream experience was (see section “2. Materials and methods” for further details). Data from DE awakenings from NREM2 sleep.

### 3.2. Controls and exploratory analysis

#### 3.2.1. Multi-channel LZC

In our previous study (Aamodt et al., 2021), instead of average single-channel LZC, we used multi-channel LZC (binarized signals were concatenated first over channels and then over time), calculated from one central and one posterior selection of 12 EEG channels (Supplementary Figure 1). To check the extent to which the choice between these two measures changed the results, we redid the analysis of the previous section, using multi-channel LZC. Results were largely the same (see Supplementary Figures 2–4 and Supplementary Tables 1–3 for details).

#### 3.2.2. LZC versus the aperiodic spectral exponent

As a comparison to a more traditional frequency-based approach, we repeated the analysis above to see how the aperiodic spectral exponent varied with sleep stage, NREM2 dream experience classification and NREM2 dream content ratings. Results were broadly similar to those for LZC, except that the estimated marginal mean spectral exponent for wake was (significantly) higher than for NREM1 (see Supplementary Figures 5–7 and Supplementary Tables 3–6 for details).

**FIGURE 6:**
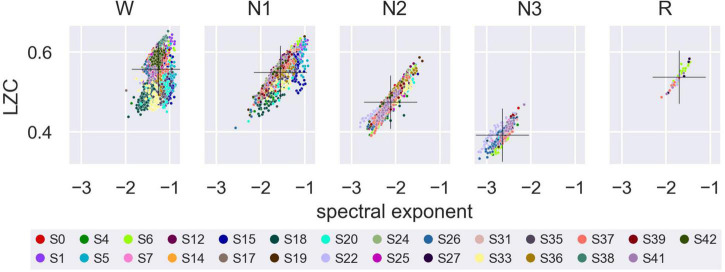
Lempel-Ziv complexity (LZC) versus the aperiodic spectral exponent within each sleep stage. Average LZC over all channels plotted against average aperiodic spectral exponent for Wake, NREM1, NREM2, NREM3, and REM sleep. Marker color indicates participant number. Overall mean LZC and overall mean aperiodic spectral exponent (calculated across participants) for each sleep stage is indicated by a black cross.

Values of LZC and the aperiodic spectral exponent were also correlated to each other, as assessed by a linear mixed model of average LZC over all channels, with average aperiodic spectral exponent over all channels as a fixed covariate, participant and trial (nested within participants) as random intercepts, and epoch as a repeated measures variable with first-order autoregressive residual variance-covariance structure (Supplementary Table 7 and Figure 6).

#### 3.2.3. Topographic patterns in single-channel LZC

**FIGURE 7:**
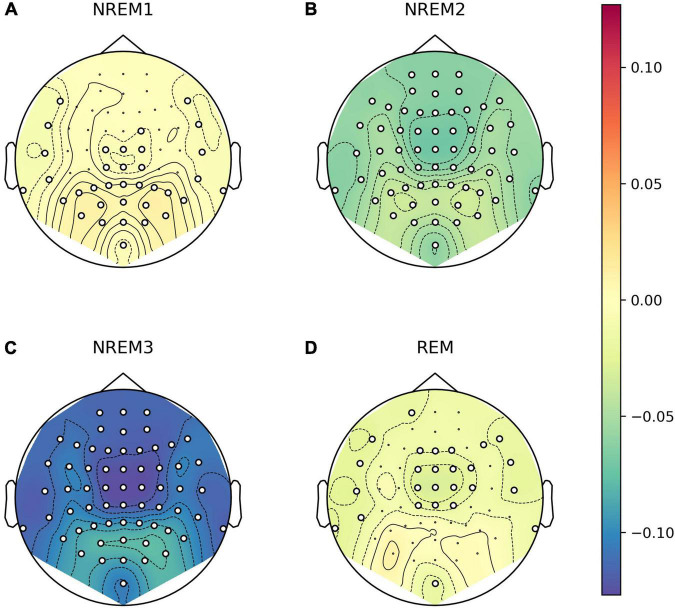
Channel-wise differences in Lempel-Ziv complexity (LZC) in different sleep stages compared to wakefulness. Topoplot of channel-wise differences in estimated marginal mean LZC for stages NREM1 **(A)**, NREM2 **(B)**, NREM3 **(C)**, and REM **(D)** versus Wake. For each EEG channel, we fit a linear mixed model (LMM) with sleep stage as a fixed factor (including intercept), and participant and trial (nested within participants) as random intercepts. Epochs were entered as repeated measures with first-order auto regressive residual variance-covariance (same as the model for average LZC over all channels as a function of sleep stage). Approximate EEG electrode positions are indicated by black dots, and channels for which LZC was significantly different from wake at the un-adjusted 0.05 level are marked by white circles.

**FIGURE 8:**
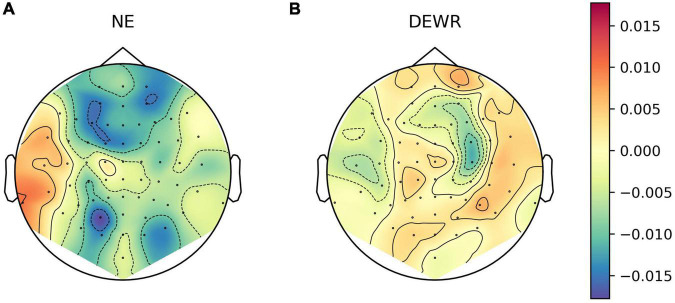
Channel-wise differences in Lempel-Ziv complexity (LZC) in NREM2 awakenings without (explicit recall of) dream experience compared to awakenings with dream experience. Topoplot of channel-wise differences in estimated marginal mean LZC for awakenings without dream experience **(A)** and awakenings without recall of dream experience contents **(B)**, versus awakenings with (explicit recall of) dream experience. For each EEG channel, we fit a linear mixed model (LLM) with dream experience classification as a fixed factor (including intercept) and participant as a random intercept (same as the model for average LZC over posterior channels as a function of dream experience classification). Approximate EEG electrode positions are indicated by black dots, and channels for which LZC was significantly different from dream experience (DE) at the un-adjusted 0.05 level are marked by white circles (there are none).

**FIGURE 9:**
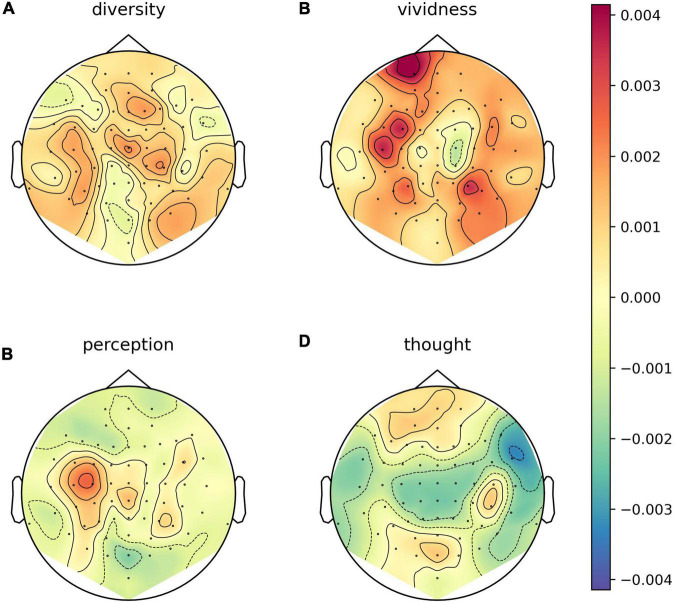
Channel-wise correlation between dream experience content ratings and Lempel-Ziv complexity (LZC) for NREM2 awakenings with dream experience. Channel-wise correlation between LZC and subjective ratings of how diverse **(A)**, vivid **(B)**, perceptual **(C)**, and thought-like **(D)** NREM2 dream experiences were. For each subjective rating, and for each EEG channel, we fit a linear mixed model (LMM) with the subjective rating of dream contents as a fixed covariate (including intercept) and participant as a random intercept. Approximate EEG electrode positions are indicated by black dots, and channels for which the subjective ratings were significant covariates at the un-adjusted 0.05 level are marked by white circles (there are none).

We explored whether there were any topographical patterns in how LZC related to sleep stage, NREM2 dreaming and NREM2 dream content ratings. While there were clear differences between wakefulness and NREM2/NREM3 sleep (Figure 7), no channels were significantly different between dreaming and non-dreaming, or correlated significantly with any of the subjective ratings of dream experience, even before any adjustment for multiple comparisons (Figures 8, 9).

#### 3.2.4. Variation in ratings of dream contents with sleep stage

**FIGURE 10:**
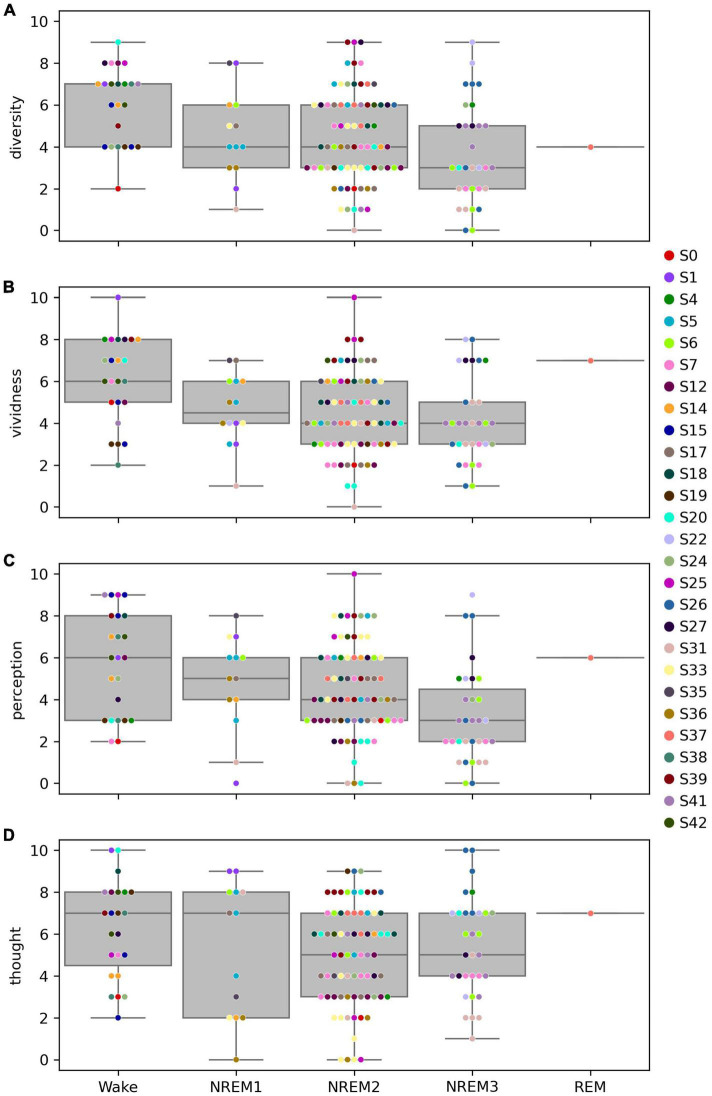
Distribution of subjective ratings of dream experience for each sleep stage. Distribution of subjective ratings of how diverse **(A)**, vivid **(B)**, perceptual **(C)**, and thought-like **(D)** dream experiences were for each of the five sleep stages (Wake, NREM1, NREM2, NREM3, and REM). Each data point corresponds to the report from one dream experience (DE) awakening. Observations are plotted on top of corresponding boxplots. Participant number is indicated by marker fill color, and observations are displaced slightly along *x*-axis to avoid overlap.

Broadly speaking, subjective ratings of how diverse, vivid, and perceptual dream contents were seemed to decrease with depth of NREM sleep, in line with expectations and consistent with the hypothesis that LZC should decrease with increasing depth of NREM sleep (distribution of ratings of how thought-like dream experiences were are included for completeness) (Figure 10).

## 4. Discussion

Sleep stage was a significant factor in the linear mixed model for between-stages variation in EEG complexity. Estimated marginal mean LZC was significantly lower for NREM2 than for wake, REM and NREM1, and significantly lower for NREM3 than for NREM2. These findings are in line with our own previous findings (Aamodt et al., 2021), as well as previous reports of how complexity of spontaneous (Shaw et al., 1999; Abásolo et al., 2015; Andrillon et al., 2016; Schartner et al., 2017b) and TMS-evoked (Casali et al., 2013) EEG signals varies between wakefulness and (deep) sleep.

The results for NREM1 were less clear. Estimated marginal mean LZC was higher for NREM1 than for wake, in contrast to expectations, but the difference was no longer significant after correcting for multiple comparisons. It is also worth noting that distinguishing between wakefulness and NREM1 during sleep scoring can be quite difficult, and hallmarks of both these sleep stages can often be present within the same 30 s sleep epoch. The contrast between wakefulness and light sleep should be investigated further, particularly since many studies on EEG complexity and sleep have focused (mainly) on deeper sleep stages.

Estimated marginal mean LZC for REM was lower than expected, although not significantly different from wake (and NREM1). There was only limited data from REM sleep, some of which stemmed from brief episodes of REM sleep within NREM sleep. As with NREM1, REM can be challenging to score accurately, which in this study was in some cases further exacerbated by poor chin-EMG signal (some of the AASM scoring rules for REM sleep relies on chin-EMG).

We failed to find any significant difference in posterior LZC between NREM2 awakenings with and without reported dream experiences, and therefore cannot reject the null hypothesis that there is no difference in posterior LZC between NREM2 awakenings with and without reported dream experience. This result is in line with negative findings from our previous study (Aamodt et al., 2021). Other studies have reported differences in TMS-evoked response between dream and non-dream awakenings from NREM sleep, indicating altered connectivity in posterior cortical areas (Nieminen et al., 2016; Lee et al., 2019) (reliable calculation of the perturbational complexity index (PCI) was not possible due to insufficient number of TMS-pulses associated with each awakening). Siclari et al. (2017) found a reduction in posterior low-frequency activity for DE awakenings relative to NE awakenings, and even found that high-frequency power in specific cortical areas (source re-constructed EEG) correlated with specific dream contents, whereas Wong et al. (2020) was unable to distinguish dreaming from non-dreaming based on power of the EEG frequency spectrum. Using a serial-awakening paradigm with immediate report, Casey et al. (2022) found that classifiers of unconsciousness and disconnectedness established by machine learning on source-reconstructed EEG data from dexmedetomidine sedation successfully generalized to propofol sedation and natural sleep (while occipital delta power did not).

It should be noted that data availability, especially the low number of NE awakenings, is a limitation of the analysis of dreaming versus non-dreaming. There are also inherent uncertainties in how accurately reported dream experience captures actual experience right before the awakenings (alarm). Although we took great care to instruct the participants, it is impossible to rule out that some dream reports may refer to earlier periods of dreaming during the sleep trial, or brief experiences during awakening, after the alarm. Indeed, we chose to exclude the question about any emotions the participant may have had right before the alarm from further analysis, because of considerable doubt as to whether the answers referred to the intended period of time. Memory failure and positivity bias may also potentially influence whether a participant reports no dream experience, dream experience without recall or dream experience with explicit recall of contents.

There was no significant correlation between posterior LZC and subjective scores of dream experience diversity and vividness. While some promising results in this direction have been reported earlier, particularly in studies of psychedelics (Schartner et al., 2017a; Timmermann et al., 2019) and “meaningfulness” of presented stimuli (Boly et al., 2015), LZC has also been found to be unresponsive to the information rate of auditory stimuli (Bola et al., 2018).

Similarly, subjective scores of how perceptual (or thought-like) the dream experience was, did not significantly correlate with posterior LZC for NREM2 awakenings with reported dream experience, meaning that we failed to reproduce the significant positive correlation that we found in our previous study (Aamodt et al., 2021).

As for reports of dreaming more generally, content ratings can in principle refer to experiences outside of the intended time period. How the scales are used can be affected by each participant’s interpretation. Even though we gave detailed written and oral explanations of the dream questionnaire, during awake practice rounds before the experiment there were still sometimes lingering misunderstandings that needed to be cleared up.

Furthermore, we cannot exclude the possibility that confounds that were not included in the analysis might in principle camouflage an actual relationship between LZC and dreaming, e.g., time from the start of the experiment, since the start of the sleep trial or since entering NREM2 sleep.

Results for both multi-channel LZC and for the aperiodic spectral exponent were mostly similar to those for single-channel LZC, except that the estimated marginal mean spectral exponent, in contrast to LZC, was higher for Wake than for NREM1.

Given the similar results between LZC and the aperiodic spectral exponent,^17^ it is natural to ask whether changes in complexity between sleep stages may just reflect changes in the slope of the power spectrum, and more broadly, whether LZC offers any practical advantage over traditional approaches to the classification of consciousness based on EEG. Indeed, the LZC of stochastic (random) signals has been found to increase with signal bandwidth (Aboy et al., 2006) and (signed) slope of the power spectrum [Supporting Information, (Toker et al., 2022)]. Furthermore, previous studies have illustrated that spectral changes may account for (a major) component of the observed difference in EEG/MEG LZC between different experimental conditions. However, some component of the difference in LZC (or any other similar measure) between datasets may also be attributed to changes in phase and in phase-spectrum interaction (Schwartzman et al., 2019; Mediano et al., 2021). Finally, both LZC and the spectral exponent could be correlated to other variables, such as excitatory-inhibitory balance (Gao et al., 2017; Lombardi et al., 2017).

From a theoretical point of view, EEG LZC may in any case be considered a more clearly motivated measure of consciousness than the slope of the EEG power spectrum, which could potentially be more directly linked to subjective experience, compared to features of the power spectrum (although it is important to keep in mind that EEG LZC itself does not directly measure complexity of neural activity either).

Exploratory analysis of topographical patterns in how LZC varies with sleep stage, NREM2 dreaming and ratings of NREM2 dream experience did not suggest that LZC for any other selection of channels was significantly different between dreaming and non-dreaming, or correlated significantly with subjective ratings of dream contents.

Ratings of how diverse, vivid, and perceptual dream experience generally seemed to decrease with depth of NREM sleep, in line with expectations and previous results (Siclari et al., 2013).

While a null result in itself does not allow us to reject the alternative hypothesis, the clear null results for every single one of the within-state tests used here suggest there may not be any (strong) relationship between (mean single-channel) LZC and (aspects of) dream experience within the same state (NREM2 sleep).

However, it is important to note the limitations mentioned above. This study should thus be seen as an incremental addition to other studies in the field. Taken together, the available evidence underscores the conclusion that much further research is needed.

## Data availability statement

The raw data supporting the conclusions of this article will be made available by the authors, without undue reservation.

## Ethics statement

The studies involving human participants were reviewed and approved by Regional Committees for Medical and Health Research Ethics–South East, Faculty of Medicine, University of Oslo (ref. 2018/1640). The patients/participants provided their written informed consent to participate in this study.

## Author contributions

AA wrote the manuscript, interpreted the results, analyzed the data, reviewed the sleep scoring, and helped collect the data and design the study. AS revised the manuscript, formulated the hypotheses statements, and helped interpret the results, collect the data, and design the study. RM revised the manuscript and sleep scored the EEG data. AK, FH, and NK revised the manuscript and helped collect the data and design the study. BT revised the manuscript, initiated the study, and helped design the study. JS revised the manuscript, formulated the hypotheses statements, helped supervise work on the manuscript, interpreted the results, initiated and designed the study, and he started the Norwegian Research Council project which this study is a part of, and he is the leader of. BJ revised the manuscript, supervised work on the manuscript, formulated the hypotheses statements, and helped interpret the results, collected the data, initiated and designed the study. All authors contributed to the article and approved the submitted version.

## Footnotes

1 Paraphrasing a global formulation of non-reductive supervenience physicalism (although this statement alone is arguably too weak to exclude substance dualism) (Internet Encyclopedia of Philosophy, 2002).

2 In the sense that changes in the physical world outside your brain alone are insufficient to allow a change in your experience. Note that there are theories of consciousness that emphasize the external environment as “its own representation,” see for example O’Regan and Noë (2001).

3 Here, the configuration of your brain should be understood to mean the full set of physical facts about the brain. The argument presented here does not tell us exactly what physical changes are relevant to conscious experience, how easy they are to observe, or how they differ from physical changes in the brain that are not (directly) relevant to conscious experience.

4 Note that while the argument above suggests that integration and differentiation may be necessary conditions for (much of) everyday human experience, it does not suggest they are sufficient, and some theories emphasize different aspects of consciousness, see for example Brown et al. (2019).

5 There exists other measures based on ongoing activity that in fact attempt to capture causal connections, see for example (Seth et al., 2006).

6 Of course, global states such as wakefulness or slow wave sleep are never truly uniform, and even within the “same” state there could be ongoing variation in brain activity that has little or no connection to conscious experience.

7 This is an important supposition that is not always made explicit. First, LZC is calculated from the binarized EEG signal (as an approximation to algorithmic complexity), and second, it is unclear to what extent signals recorded from the outside of a system should be expected to reflect the internal dynamics of that system. Arguably, the second part of this issue goes beyond both consciousness studies and EEG complexity measures, see for example (Jonas and Kording, 2017).

8 For the first experiment, the EO/EC recordings were done with researchers in the room and the participant (S20) sitting up.

9 We tried to allow at least 1 min of NREM2 sleep before awakening, but sometimes woke up the participant in other sleep stages because they had trouble sleeping, or because they quickly transitioned to NREM3 sleep.

10 For S20 we recorded additional EO and EC trials at the end of the experiment.

11 To avoid saturation of channels, the recording was not always started immediately at the beginning of each sleep trial (for some trials, it was still necessary to perform DC correction after starting the recording).

12 Participants sometimes fell asleep during the EC trials, or had trouble sleeping during sleep trials, which is why some EC reports are from NREM1/NREM2, and some sleep trial reports are from a period of wakefulness.

13 Note that *a* = *abs*(*hilbert*(*x*)) in the referenced figure refers to the amplitude of the complex valued analytical representation of the EEG signal. The notation in the figure is consistent with the naming of SciPy’s *hilbert* function, which despite its name, returns the analytical signal, and not the Hilbert transform of the EEG signal (SciPy, 2022).

14 See Schwartzman et al. (2019) for an approach to assess how much of the difference in EEG LZC between conditions is associated with spectral changes (or phase changes), and Mediano et al. (2021) for a general decomposition of the difference in some observable between two time series datasets into a component associated with spectral changes, a component associated with with phase changes and a component associated with changes in phase-spectrum interaction.

15 In this study we used LMMs, rather than the more complicated generalized linear mixed models (GLMMs) used in Aamodt et al. (2021), because there was not much of a problem with LZC values close to 1.0 (we did however check that the different choice of models did not matter to the results of the analysis of posterior multichannel LZC as a function of dream experience classification and ratings).

16 Replacing ratings of how perceptual the dream was by the difference between perception and thought ratings gave similar results.

17 We did not analyze the relationship between multi-channel LZC and the aperiodic spectral exponent (or between single-channel LZC and multi-channel LZC), but see Supplementary Figures 9, 10, and results from Schartner et al. (2017b), which indicates that changes in multi-channel complexity measures did not only reflect spectral changes.
