# Supplementary material for: EEG Lempel-Ziv complexity varies with sleep stage, but does not seem to track dream experience
Source: Front Hum Neurosci. 2023 Jan 10;16:987714. doi: 10.3389/fnhum.2022.987714 (PMC9871639; doi:10.3389/fnhum.2022.987714)
Supplement: Supplementary file 1 [file Data_Sheet_1.pdf]

Supplementary Material

1 Supplementary Data

1.1 Instructions and questionnaire sent to participants

1.1.1 Introduction

On the day of the sleep experiment, you will be sleeping in the lab while we record your brain signals. Every once in a while, you will be awakened by an alarm sound, and we will ask you about any (dream) experiences you might have had right before the alarm. The questions will be the same for each awakening (see questionnaire on next page).

However, when you are drowsy and confused right after waking up, it can be very hard to understand and answer even quite simple questions. Therefore it is important to make sure that you understand all the questions beforehand (ask if something is unclear), and practice answering them at home the last week before the experiment.

We want all participants to do approximately the same preparations before sleeping in the lab, to minimize confounding variation in our data. We therefore ask that you do the following (and tell us if you deviated from these instructions):

1. Read through the questions and their explanations. Make sure you understand everything and ask us if anything is unclear.
2. Practice answering the dream questionnaire a few times while you are awake.
3. You should also try to answer the questionnaire immediately after waking up the last few mornings before the experiment, and particularly after waking up on the day of the experiment. We suggest that you set an alarm to wake you up in the morning (maybe use a different sound than what you are used to). Immediately after waking up, answer the questionnaire for yourself, just like you would while being interviewed in the lab (if possible, say the answer out loud and clear). Please try to follow the instructions and explanations for the questions as closely as you can.

Please note: The only thing important to the experiment is that you describe your experience faithfully, and we do not necessarily expect you to always, or even once, report an experience. If the most faithful description is "there was nothing", then that is what you should report.

1.1.2 Questionnaire

<u>Question 1:</u> *What was the last thing going through your mind?*
<u>Norwegian:</u> *Hva var det siste som gikk gjennom hodet ditt?*
<u>Short form:</u> *Last experience?*

Describe the most recent experience (for example image, thought or emotion) which you had before the alarm sound (but after falling asleep), *if you experienced anything at all - it is just as fine if you didn't experience anything*. By experience we mean "any kind of mental activity", including thoughts, dreams, perceptions, emotions, etc (e.g. if asked while being awake the last thing going

through your mind might just be the scene you see in front of you). Take your time and describe your experience as completely as you can. We will stop you if we need to go on to the next question. If you feel like you experienced something right before the alarm, but you can't remember the contents of your experience, please just say something like "there was something, but I don't remember".

There are no wrong or right answers, and your description could for example include:

- no experience or just a feeling of having experienced something without any memory of the experience
- emotions or feelings you were having
- sensations or perceptions (seeing, hearing, smelling, feeling, tasting, bodily sensations etc.)
- features (shape, color, size, texture, etc.)
- objects and scenery that were part of your experience (people, animals, inanimate objects, rooms, landscapes, etc.)
- movement, change, activities and events (what was going on and who/what was doing what)
- your own role in the experience (actively participating, only observing)
- awareness that you were experiencing something
- your experience of self
- narrative structure of the experience
- thoughts or reflections
- intentions, plans
- awareness that you were asleep and that your experience was a dream
- etc, etc …

<u>Question 2:</u> *What was the last emotion you had?*
<u>Norwegian:</u> *Hva var den siste følelsen du hadde?*
<u>Short form:</u> *Last emotion?*

Describe your most recent feelings or emotions (for example anger, disgust, fear, happiness, sadness, surprise etc.) which you had before the alarm sound (but after falling asleep), *if you experienced any emotions at all - it is just as fine if you didn't experience any emotions*. These emotions might be connected to the experience you described under Question 1, but they don't have to be. You may also be repeating some of your answer to Question 1, but that is completely fine, just answer both questions as fully as possible.

<u>Question 3:</u> *How diverse was your experience on a scale from 0-10?*
<u>Norwegian:</u> *Hvor variert var din opplevelse på en skala fra 0-10?*
<u>Short form:</u> *Diversity 0-10?*

Please rate your experience from 0-10, where 0 means "I did not experience anything at all" and 10 means "my experience was just as diverse or more diverse than my normal waking experience". The more different emotions, sensory impressions, objects, actions, thoughts, your experience consists of, the more diverse (rich, varied) we consider the experience to be. A minimally diverse experience could thus be something like a visual image filled with a single uniform color, for example. *Note that diversity applies not only to perception but also to thoughts and emotions, i.e. we could talk about a simple idea of a single concept as opposed to complex reasoning, or a singular fear as opposed to the complex mixture of emotions you might have while starting university.*

Question 4: *How vivid was your experience on a scale from 0-10?*
Norwegian: *Hvor klar var din opplevelse på en skala fra 0-10?*
Short form: *Vividness 0-10?*

Please give a rating of your experience from 0-10, where 0 means "I did not experience anything at all" and 10 means "my experience was just as vivid or more vivid than my normal waking experience". The clearer and more life-like your experience, the more vivid (as opposed to blurry and diffuse) we consider the experience to be. *Unlike Question 3, we are not here asking about the diversity of your experience.* A very vivid experience can thus be anything from a very clear perception of the color red to a life-like experience of a garden party. On the other hand, a minimally vivid experience of the same could be a very vague perception or thought of something reddish or a very blurry impression of an outdoor scene. *Also, note that vividness applies not only to perception but also to thoughts and emotions, i.e. we often talk about a vague idea as opposed to clear understanding, or unclear feeling of unrest as opposed to distinct fear.*

Question 5: *How perceptual was your experience on a scale from 0-10?*
Norwegian: *Hvor perseptuell var din opplevelse på en skala fra 0-10?*
Short form: *Perceptual 0-10?*

Please rate the extent to which your experience was perceptual in character. In this context, perceptual refers to any visual, auditory, olfactory or bodily experience/imagery that you had.

Question 6: *How thought-like was your experience on a scale from 0-10?*
Norwegian: *Hvor tanke-aktig var din opplevelse på en skala fra 0-10?*
Short form: *Thought-like 0-10?*

Please rate how thought-like your experience was. Thought-like refers to reasoning or other mental processes not necessarily related to/accompanied by perceptual experiences/imagery (or inner speech). A typical example of an experience report from a participant that was very thought-like could be the following: *"I was thinking of tax exemption, and it skipped around to something else ... one of the points made in the class ... was that you had to provide over half of the support of a person in order to claim them as dependents."* (Foulkes, 1962)

2 Supplementary Figures and Tables

2.1 Supplementary Figures

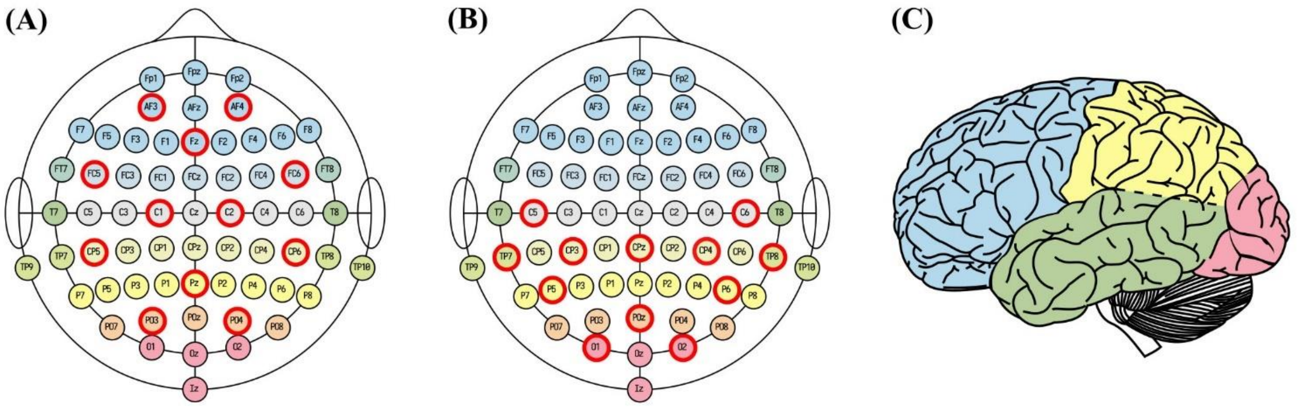

Supplementary Figure 1. EEG channels for calculation of multi-channel LZC. Central **(A)** and posterior **(B)** channel selections for calculation of multi-channel LZC. Electrode fill color indicates associated cortical lobe **(C)** [adapted from illustration by Laurens R. Krol, distributed under a CC0 1.0 license (Krol, 2020)].

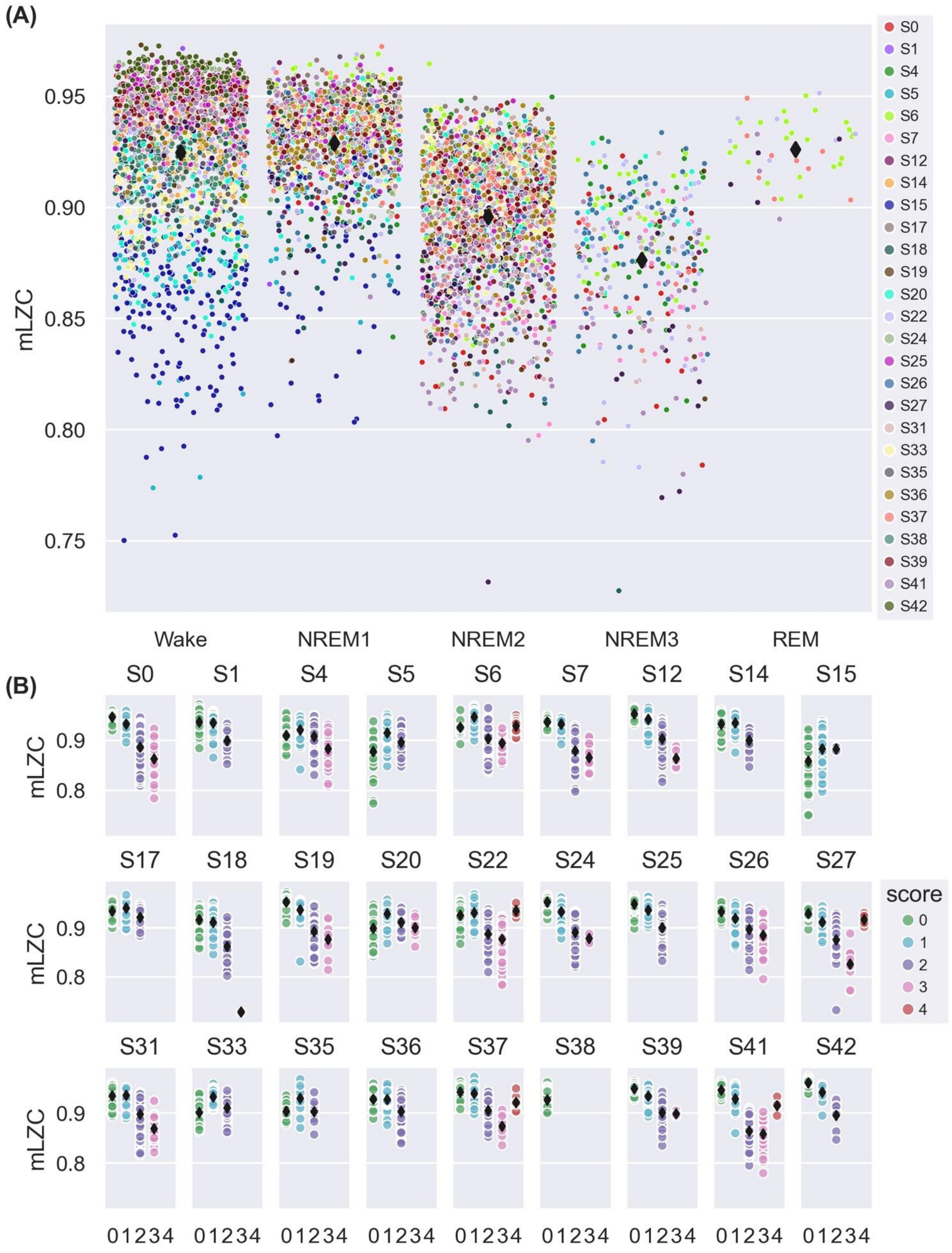
(A)
mLZC
0.95
0.90
0.85
0.80
0.75
Wake
NREM1
NREM2
NREM3
REM
S0
S1
S4
S5
S6
S7
S12
S14
S15
S17
S18
S19
S20
S22
S24
S25
S26
S27
S31
S33
S35
S36
S37
S38
S39
S41
S42
(B)
S0
S1
S4
S5
S6
S7
S12
S14
S15
S17
S18
S19
S20
S22
S24
S25
S26
S27
S31
S33
S35
S36
S37
S38
S39
S41
S42
mLZC
0.9
0.8
score
0
1
2
3
4
01234

Supplementary Figure 2. Variation in (central) multi-channel LZC with sleep stage. (A) Multi-channel LZC vs. sleep stage for the central channel selection (**Supplementary Figure 1A**). Each data point corresponds to one 30s sleep epoch. Observations are randomly scattered along the x-axis to reduce overlap, and participant number is indicated by marker fill color. Overall mean values for each sleep stage are indicated by black diamond markers. **(B)** Central multi-channel LZC vs. sleep stage (0 = W, 1 = NREM1, 2 = NREM2, 3 = NREM3, 4 = REM), plotted separately for each study participant. Each data point corresponds to one 30s sleep epoch. Fill color indicates sleep stage, and black diamond markers indicate participant mean values for each stage.

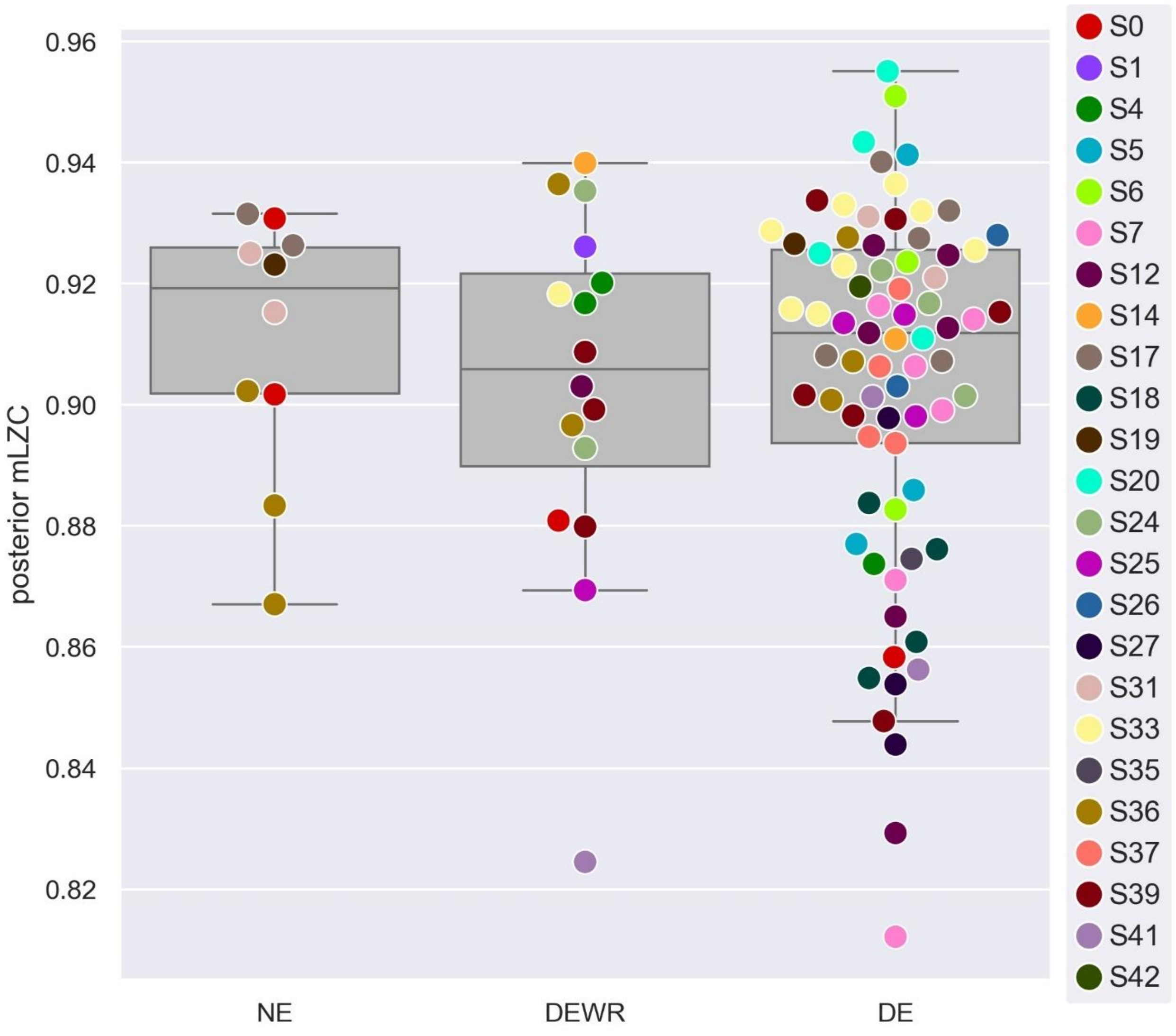

Supplementary Figure 3. Posterior multi-channel LZC versus NREM2 dream experience classification. Posterior multi-channel LZC vs. NREM2 dream experience class (NE = no experience, DEWR = dream experience without recall of contents, DE = dream experience), for the posterior EEG channel selection (**Supplementary Figure 2B**). Each data point corresponds to the last 30s sleep epoch before an awakening from NREM2 sleep. Observations are plotted on top of corresponding boxplots. Participant number is indicated by marker fill color, and observations are displaced slightly along x-axis to avoid overlap.

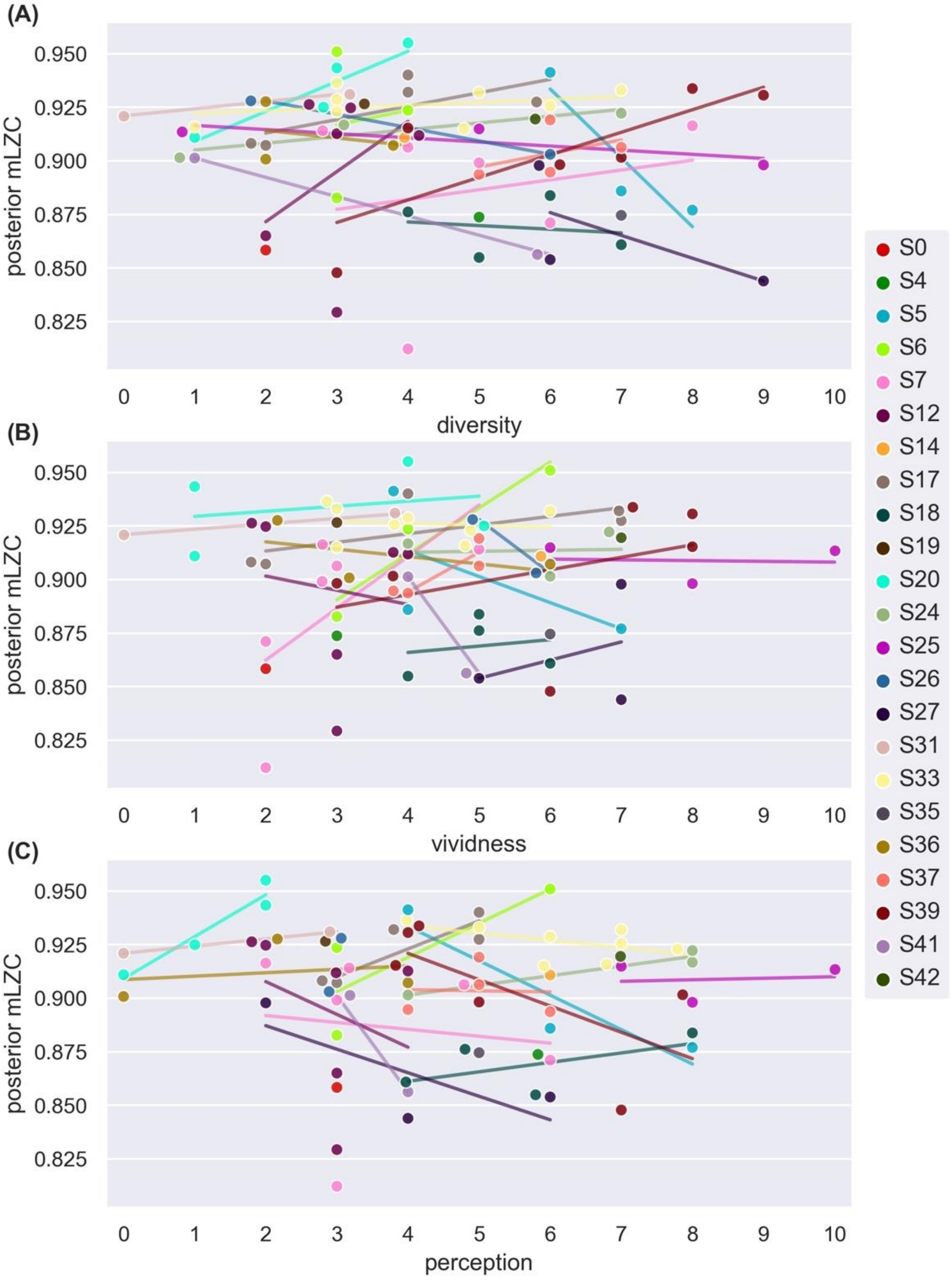

Supplementary Figure 4. Posterior multi-channel LZC versus subjective ratings of NREM2 dream experience. Posterior multi-channel LZC vs. ratings of how diverse **(A)**, vivid **(B)** and perceptual **(C)** NREM2 dream experience was. Each data point corresponds to the last 30s sleep epoch before an awakening from NREM2 sleep. Participant number is indicated by marker fill color, and observations are displaced slightly along x-axis to avoid overlap. Linear trend for each participant (for which there is more than one data point) is indicated by background line segments (visual aid only).

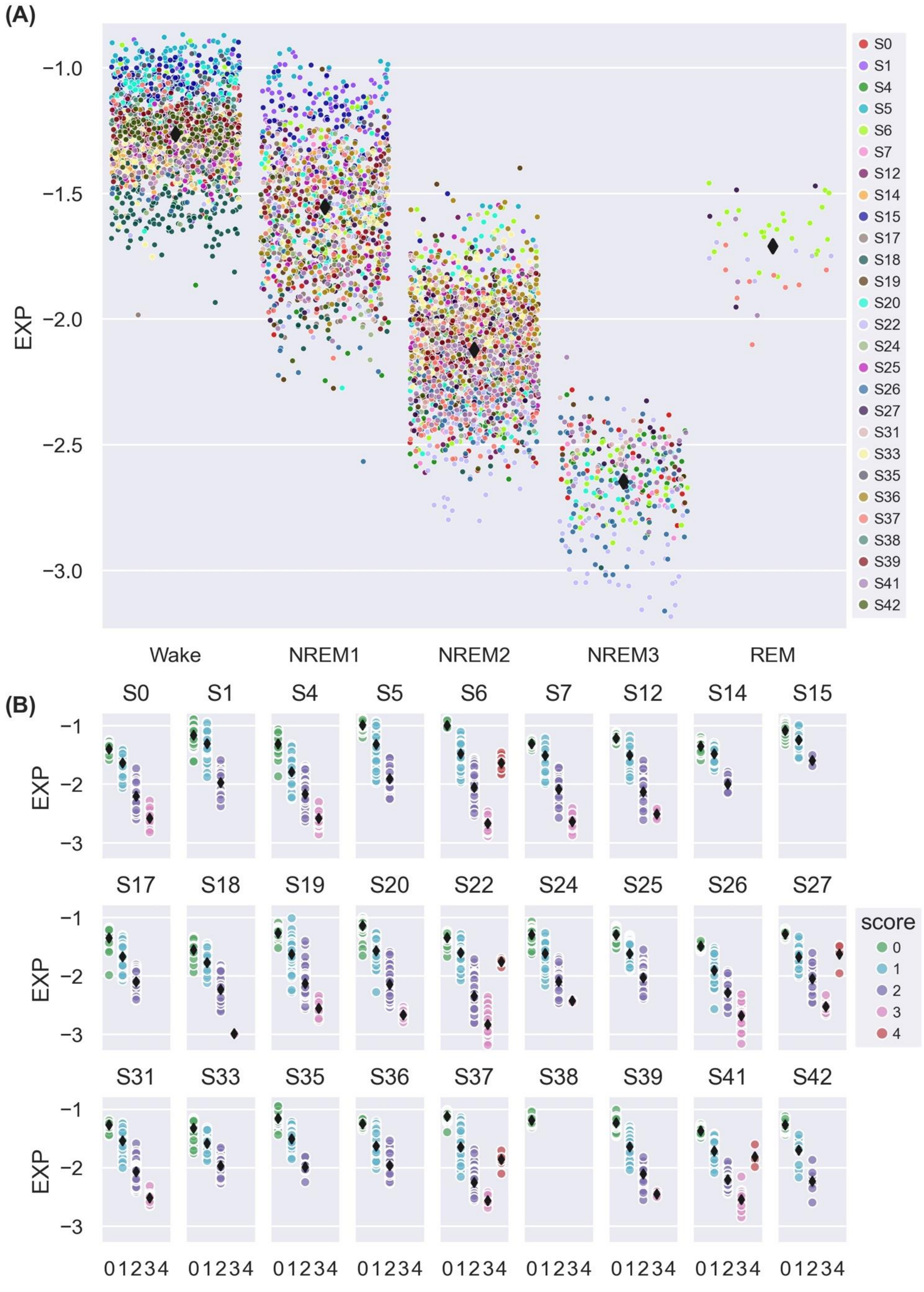
(A)
EXP
−1.0
−1.5
−2.0
−2.5
−3.0
Wake
NREM1
NREM2
NREM3
REM
S0
S1
S4
S5
S6
S7
S12
S14
S15
S17
S18
S19
S20
S22
S24
S25
S26
S27
S31
S33
S35
S36
S37
S38
S39
S41
S42
(B)
score
0
1
2
3
4
01234

Supplementary Figure 5. Variation in average whole-brain aperiodic spectral exponent with sleep stage. (A) Average aperiodic spectral exponent vs. sleep stage for the whole-brain channel selection (all channels, see **Figure 2A**). Each data point corresponds to one 30s sleep epoch. Observations are randomly scattered along the x-axis to reduce overlap, and participant number is indicated by marker fill color. Overall mean values for each sleep stage are indicated by black diamond markers. **(B)** Average whole-brain aperiodic spectral exponent vs. sleep stage (0 = W, 1 = NREM1, 2 = NREM2, 3 = NREM3, 4 = REM), plotted separately for each study participant. Each data point corresponds to one 30s sleep epoch. Fill color indicates sleep stage, and black diamond markers indicate participant mean values for each stage.

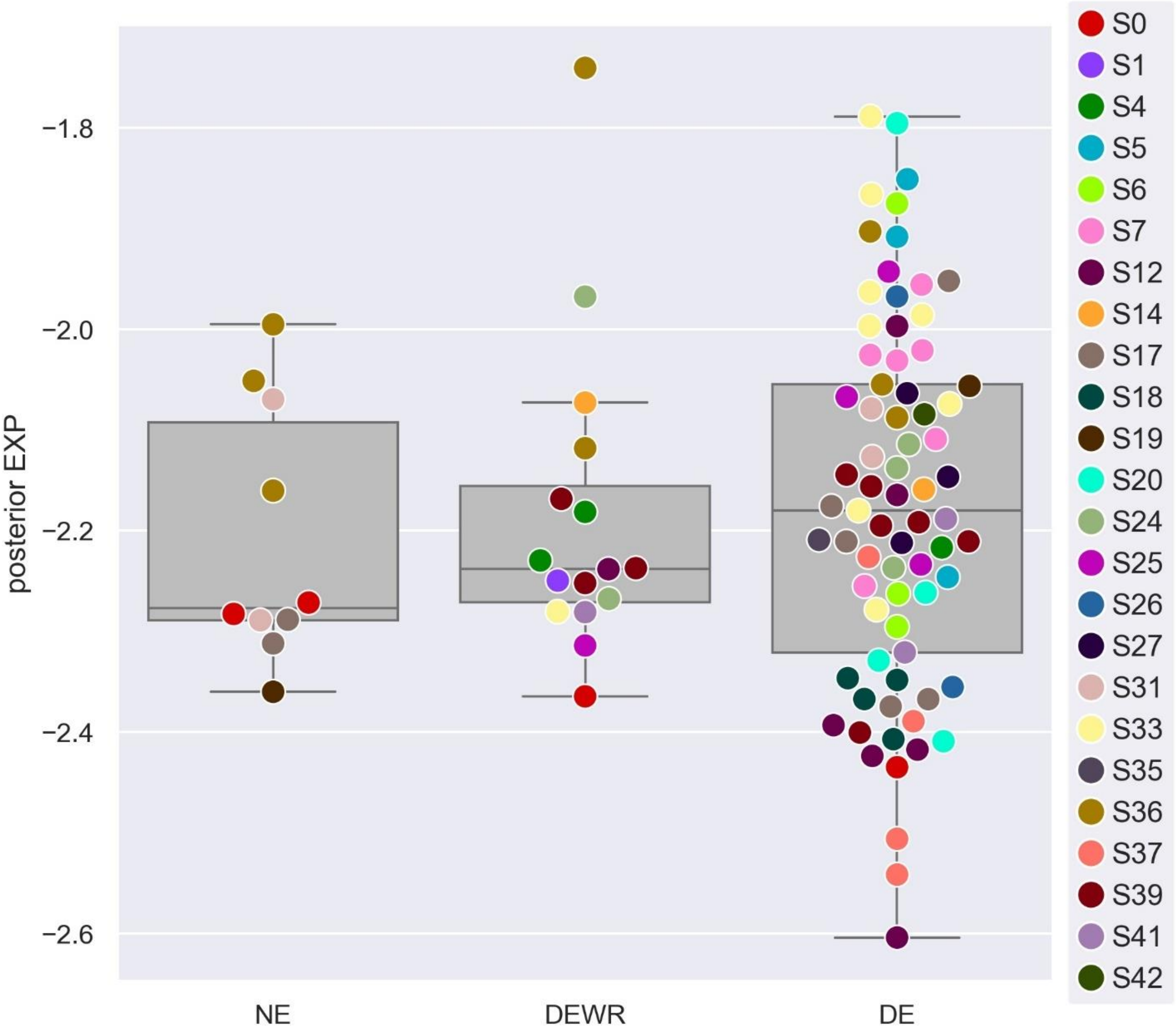

Supplementary Figure 6. Average posterior aperiodic spectral exponent versus NREM2 dream experience classification. Average aperiodic spectral exponent vs. NREM2 dream experience class (NE = no experience, DEWR = dream experience without recall of contents, DE = dream experience), for the posterior EEG channel selection (see **Figure 2B**). Each data point corresponds to the last 30s sleep epoch before an awakening from NREM2 sleep. Observations are plotted on top of corresponding boxplots. Participant number is indicated by marker fill color, and observations are displaced slightly along x-axis to avoid overlap.

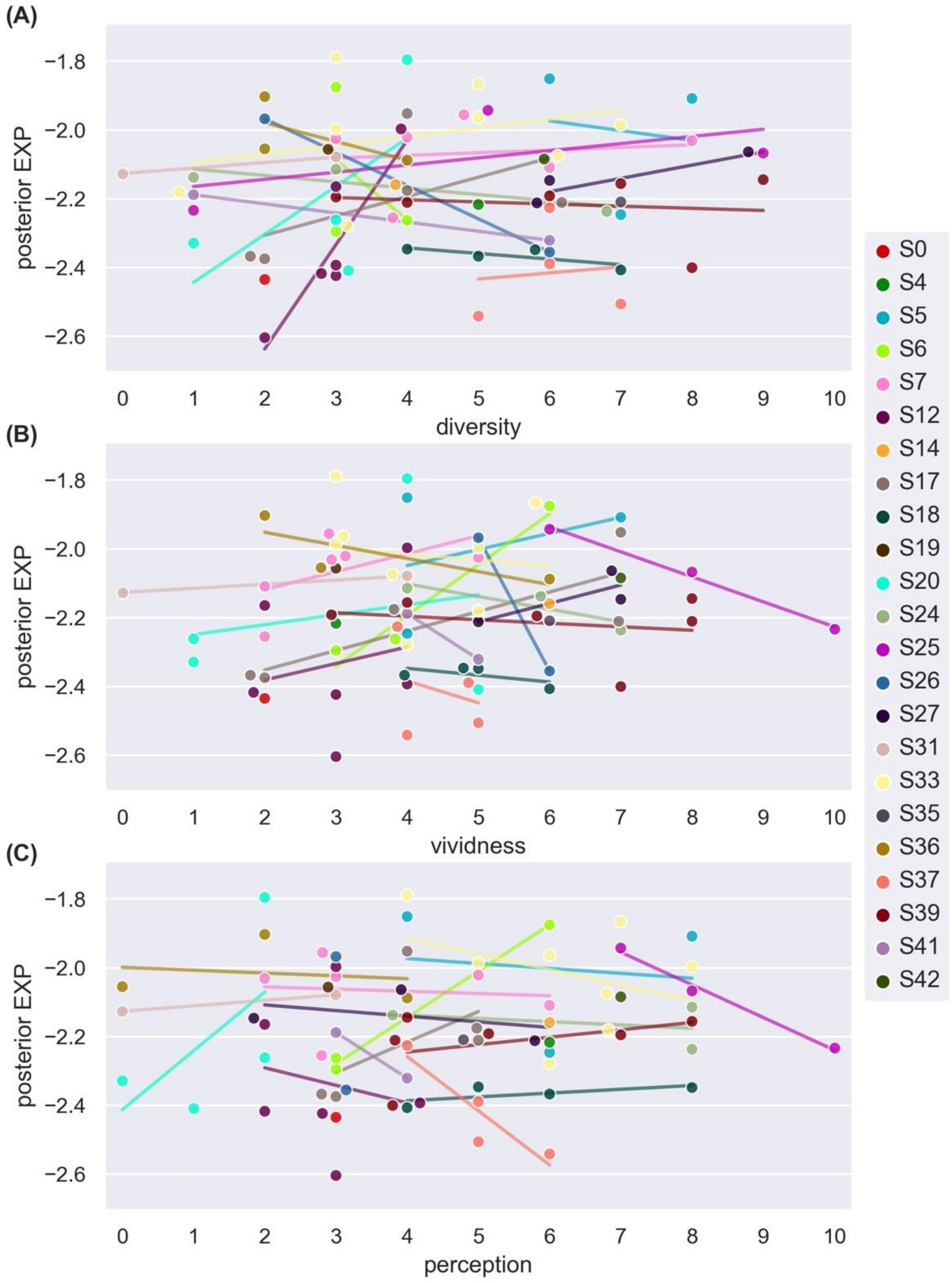

Supplementary Figure 7. Average posterior aperiodic spectral exponent versus subjective ratings of NREM2 dream experience. Average posterior aperiodic spectral exponent vs. ratings of how diverse **(A)**, vivid **(B)** and perceptual **(C)** NREM2 dream experience was. Each data point corresponds to the last 30s sleep epoch before an awakening from NREM2 sleep. Participant number is indicated by marker fill color, and observations are displaced slightly along x-axis to avoid overlap. Linear trend for each participant (for which there is more than one data point) is indicated by background line segments (visual aid only).

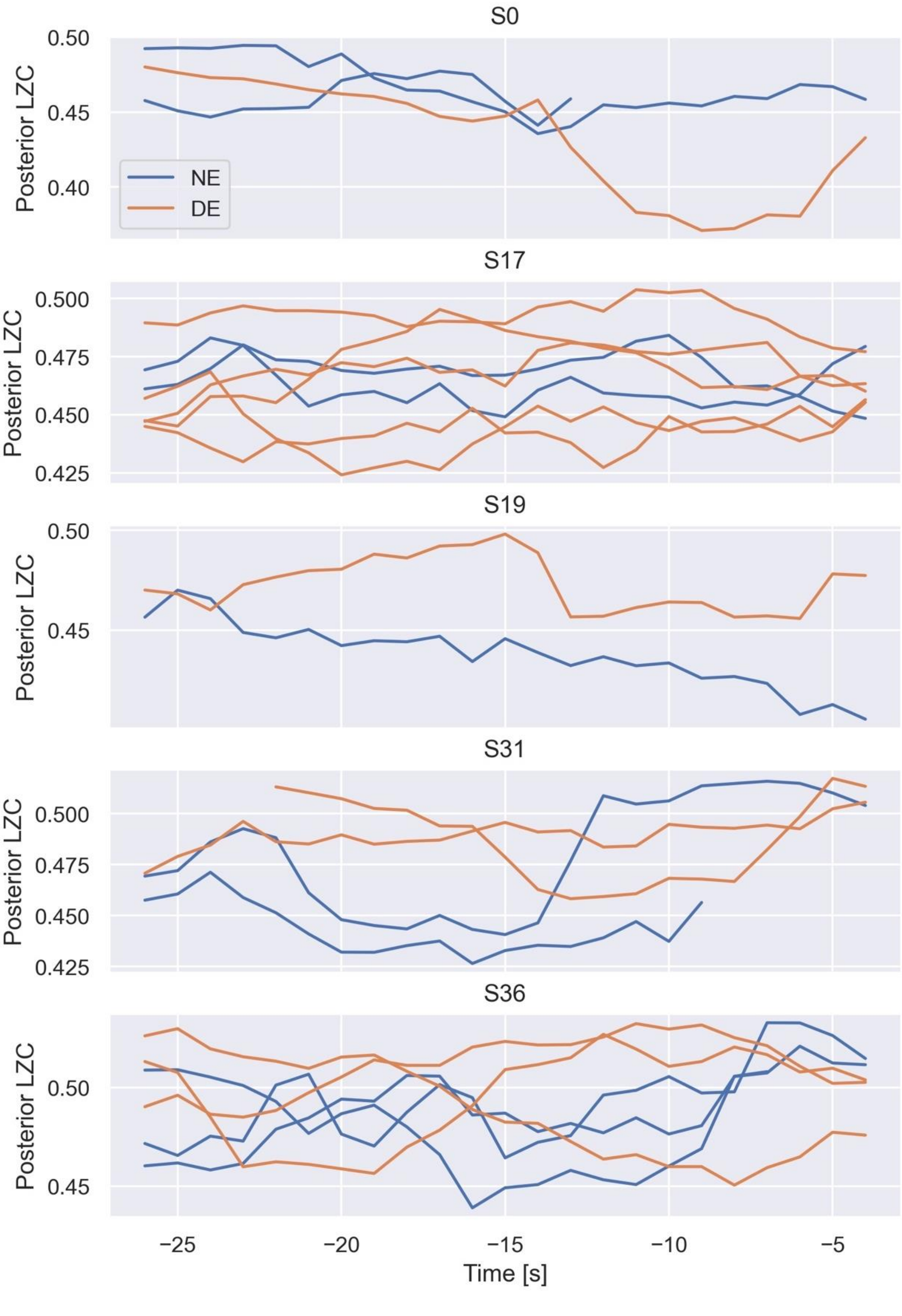

Supplementary Figure 8. Average posterior single-channel LZC versus time for the last sleep epoch before NE and DE awakenings from NREM2 sleep. Posterior LZC vs. time (center of the calculation window) for the five participants (S0, S17, S19, S31, S36) that had both NE and DE awakenings from NREM2 sleep. The duration of the calculation windows was 8 s, with 7 s overlap. LZC is missing for some of the calculation windows due to excluded stretches of EEG data.

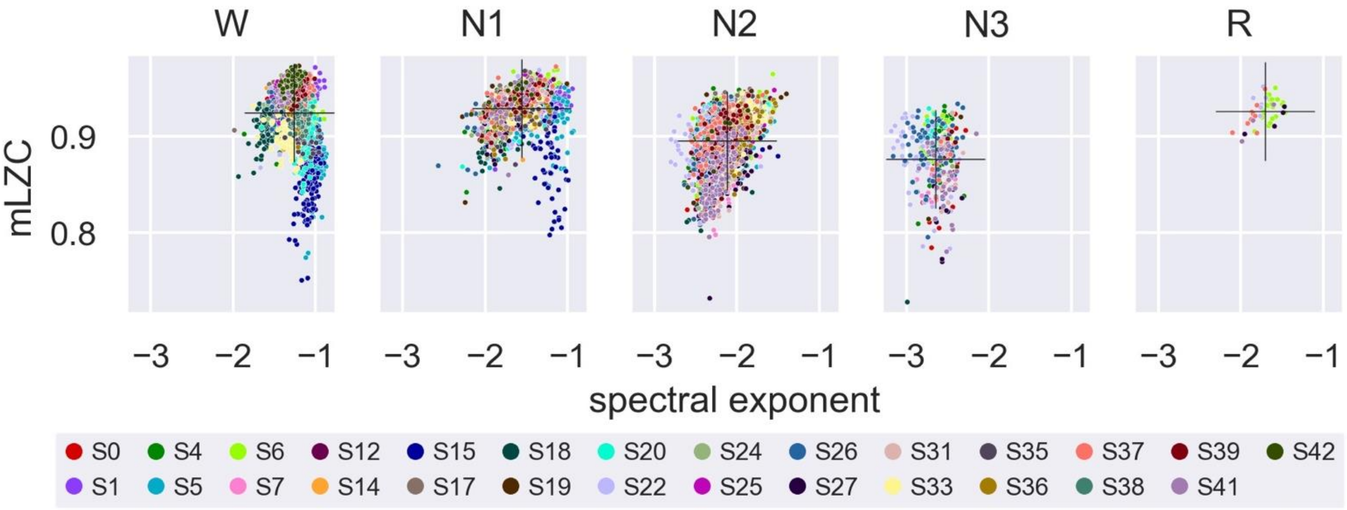

Supplementary Figure 9. (Central) multi-channel LZC versus the aperiodic spectral exponent within each sleep stage. Multi-channel LZC (calculated from the central channel selection in Supplementary Figure 1A) plotted against average aperiodic spectral exponent for Wake, NREM1, NREM2, NREM3 and REM sleep. Marker color indicates participant number. Overall mean multi-channel LZC and overall mean aperiodic spectral exponent (calculated across participants) for each sleep stage is indicated by a black cross.

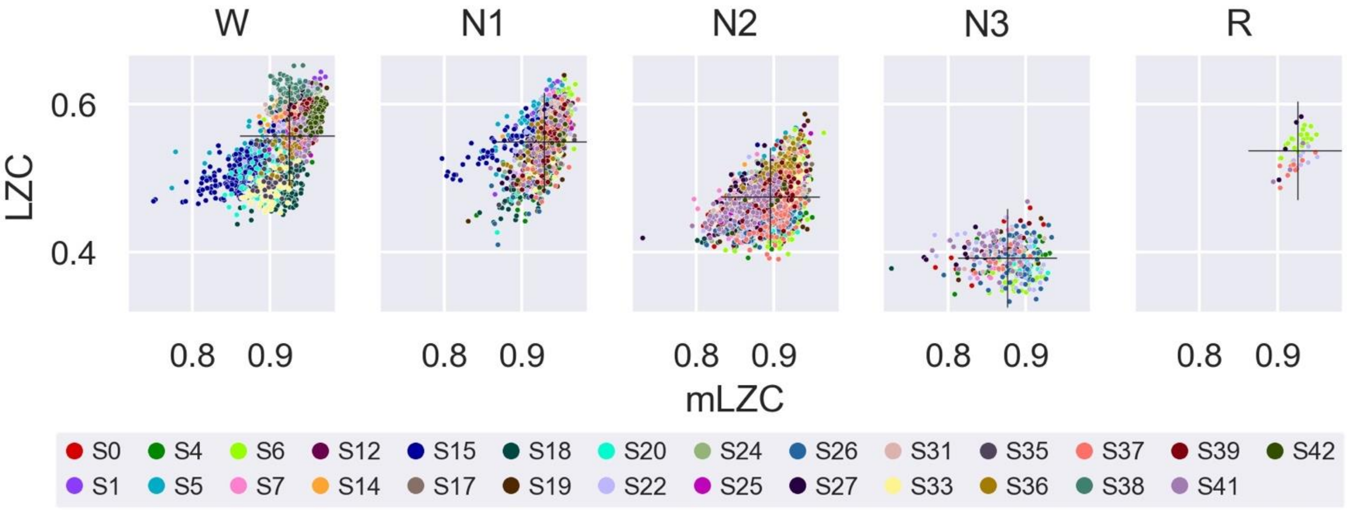

Supplementary Figure 10. Whole-brain average single-channel LZC versus (central) multi-channel LZC. Whole-brain average single-channel LZC plotted against multi-channel LZC (calculated from the central channel selection in Supplementary Figure 1A) for Wake, NREM1, NREM2, NREM3 and REM sleep. Marker color indicates participant number. Overall mean single-channel LZC and overall mean multi-channel LZC (calculated across participants) for each sleep stage is indicated by a black cross.

2.2 Supplementary Tables

Supplementary Table 1: (Central) multi-channel LZC as function of sleep stage. Summary of results from linear mixed model (LMM) of central multi-channel LZC as a function of sleep stage

**Response**	**Fixed effect**	**df1**	**df2**	**F**	**sig.**					
**mLZC**	*intercept*	1	28	82553	<.0001					
	sleep stage	4	2716	457	<.0001		**sig. (pairwise comparison)**			
	**EMMs**	**estimate**	**std. err.**	**CI_{lower}**	**CI_{upper}**		*NREM1*	*NREM2*	*NREM3*	*REM*
	wake	.927	.003	.920	.933		.047	<.001	<.001	.347
	NREM1	.929	.003	.922	.935			<.001	<.001	.609
	NREM2	.896	.003	.890	.903				<.001	<.001
	NREM3	.875	.003	.868	.882					<.001
	REM	.931	.005	.921	.941					
	**Random effect covariance**		**estimate**	**std. err.**	**Z**	**sig.**	**CI_{lower}**	**CI_{upper}**		
	variance (participant)		2.33e-4	7.50e-5	3.11	.002	1.24e-4	4.38e-4		
	*variance (participant * trial)*		1.55e-4	2.00e-5	7.74	<.001	1.20e-4	2.00e-4		

Supplementary Table 2. Posterior multi-channel LZC as a function of NREM2 dream experience class. Summary of results from a linear mixed model (LLM) of posterior multi-channel LZC as a function of dream experience class, for awakenings from NREM2 sleep.

**Response**	**Fixed effect**	**df1**	**df2**	**F**	**sig.**				
**$mLZC_{posterior}$**	*intercept*	1	35.2	32892	<.001				
	experience class	2	92.5	.055	.946			**sig. (pairwise comparison)**	
	**EMMs**	**estimate**	**std. err.**	**CI_{lower}**	**CI_{upper}**			*DEWR*	*DE*
	NE	.907	.009	.888	.926			.742	.783
	DEWR	.903	.007	.888	.918				.890
	DE	.904	.004	.895	.913				
	**Random effect covariance**		**estimate**	**std. err.**	**Z**	**sig.**	**CI_{lower}**	**CI_{upper}**	
	variance (participant)		2.34e-4	1.26e-4	1.86	.063	8.16e-5	6.72e-4	

Supplementary Table 3. Posterior multi-channel LZC as a function of subjective ratings of NREM2 dream contents. Summary of results from a linear mixed model (LLM) of posterior multi-channel LZC as a function of subjective ratings of dream experience vividness and richness, and a similar model of posterior multi-channel LZC as a function of ratings of how perceptual dream experience was. Data from DE awakenings from NREM2 sleep.

**Response**	**Fixed effect**	**df1**	**df2**	**F**	**sig.**	**estimate**	**std. err.**	**t**	**CI_{lower}**	**CI_{upper}**
**$mLZC_{posterior}$**	*diversity*	1	68.4	.530	.469	-.0012	.0017	-.728	-.0046	.0022
	vividness	1	70.0	1.104	.297	.0020	.0019	1.051	-.0018	.0059
	(intercept)	1	55.9	7510	<.001	.8996	.0104	86.7	.8788	.9204
	**Random effect covariance**		**estimate**	**std. err.**	**Z**	**sig.**	**CI_{lower}**	**CI_{upper}**		
	variance (participant)		.0002	.0001	1.46	.144	5.63e-5	.0008		

**Response**	**Fixed effect**	**df1**	**df2**	**F**	**sig.**	**estimate**	**std. err.**	**t**	**CI_{lower}**	**CI_{upper}**
**$mLZC_{posterior}$**	*perception*	1	60.5	.907	.345	-.0166	.0175	-.952	-.0515	.0183
	(intercept)	1	47.5	10219	<.001	.9108	.0090	101.1	.8926	.9289
	**Random effect covariance**		**estimate**	**std. err.**	**Z**	**sig.**	**CI_{lower}**	**CI_{upper}**		
	variance (participant)		.0002	.0001	1.67	.095	7.14e-5	.0007		

Supplementary Table 4: Whole-brain average aperiodic spectral exponent as function of sleep stage. Summary of results from linear mixed model (LMM) of whole-brain average aperiodic spectral exponent as a function of sleep stage.

Response	Fixed effect	df1	df2	F	sig.					
EXP	*intercept*	1	28	3838	<.001					
	sleep stage	4	2806	1316	<.001		sig. (pairwise comparison)			
	**EMMs**	**estimate**	**std. err.**	**CI_{lower}**	**CI_{upper}**		*NREM1*	*NREM2*	*NREM3*	*REM*
	wake	-1.386	.028	-1.444	-1.328		<.001	<.001	<.001	<.001
	NREM1	-1.593	.028	-1.651	-1.535			<.001	<.001	.011
	NREM2	-1.984	.028	-2.042	-1.925				<.001	<.001
	NREM3	-2.302	.030	-2.364	-2.240					<.001
	REM	-1.690	.047	-1.782	-1.598					
	**Random effect covariance**		**estimate**	**std. err.**	**Z**	**sig.**	**CI_{lower}**	**CI_{upper}**		
	variance (participant)		.0185	.0060	3.10	.002	.0099	.0348		
	*variance (participant * trial)*		.0136	.0021	6.49	<.001	.0101	.0184		

Supplementary Table 5. Posterior average aperiodic spectral exponent as a function of NREM2 dream experience class. Summary of results from linear mixed model (LLM) of posterior average aperiodic spectral exponent LZC as a function of dream experience class for awakenings from NREM2 sleep. The data for the DEWR category contained an outlier (see Supplementary Figure 6). Excluding this data point gave estimated marginal mean posterior aperiodic spectral exponent for DEWR between the values for NE and DE, but results were otherwise similar.

Response	Fixed effect	df1	df2	F	sig.				
$EXP_{posterior}$	*intercept*	1	41.7	5550	<.001				
	experience class	2	93.3	.617	.542			sig. (pairwise comparison)	
	**EMMs**	**estimate**	**std. err.**	**CI_{lower}**	**CI_{upper}**			*DEWR*	*DE*
	NE	-2.236	.056	-2.347	-2.125			.526	.283
	DEWR	-2.194	.044	-2.282	-2.107				.666
	DE	-2.175	.027	-2.230	-2.121				
	**Random effect covariance**		**estimate**	**std. err.**	**Z**	**sig.**	**CI_{lower}**	**CI_{upper}**	
	variance (participant)		.00830	.0039	2.13	.033	.0033	.0208	

Supplementary Table 6. Posterior average aperiodic spectral exponent versus subjective ratings of NREM2 dream contents. Summary of results from a linear mixed model (LLM) of posterior average aperiodic spectral exponent as a function of subjective ratings of dream experience vividness and richness, and a similar model of posterior average aperiodic spectral exponent as a function of ratings of how perceptual dream experience was. Data from NREM2 DE awakenings.

Response	Fixed effect	df1	df2	F	sig.	estimate	std. err.	t	CI_{lower}	CI_{upper}
$EXP_{posterior}$	*diversity*	1	68.5	.199	.731	.0036	.0105	.346	-.0173	.0245
	vividness	1	69.9	.802	.374	.0107	.0120	.895	-.0132	.0347
	(intercept)	1	60.5	1211	<.001	-2.24	.0644	-34.8	-2.369	-2.111
	**Random effect covariance**		**estimate**	**std. err.**	**Z**	**sig.**	**CI_{lower}**	**CI_{upper}**		
	variance (participant)		.0091	.0049	1.85	.065	.0031	.0262		

Response	Fixed effect	df1	df2	F	sig.	estimate	std. err.	t	CI_{lower}	CI_{upper}
$EXP_{posterior}$	*perception*	1	61.8	.185	.668	.0465	.108	.430	-.1694	.2623
	(intercept)	1	50.0	1555	<.001	-2.196	.056	-39.4	-2.308	-2.084
	**Random effect covariance**		**estimate**	**std. err.**	**Z**	**sig.**	**CI_{lower}**	**CI_{upper}**		
	variance (participant)		.0087	.0049	1.79	.073	.0029	.0260		

Supplementary Table 7: Average LZC as a function of average aperiodic spectral exponent. Summary of results from a linear mixed model (LMM) of average single-channel LZC over all channels as a function of average aperiodic spectral exponent over all channels.

**Response**	**Fixed effect**	**df1**	**df2**	**F**	**sig.**	**estimate**	**std. err.**	**t**	**CI_{lower}**	**CI_{upper}**
**LZC**	*EXP*	1	3240	9870.2	<.001	.1138	.001	99.3	.7014	.7223
	(intercept)	1	33.6	19054.4	<.001	.7119	.005	138.0	.1115	.1160
	**Random effect covariance**		**estimate**	**std. err.**	**Z**	**sig.**	**CI_{lower}**	**CI_{upper}**		
	variance (participant)		.0006	.0002	3.34	<.001	.00033	.00105		
	variance (participant ∗ trial)		.0002	2.8e-5	6.35	<.001	.00013	.00025		

Supplementary Table 8. Per-subject posterior average LZC as a function of NREM2 dream experience class. Summary of results from per-subject linear mixed models (LLMs) of posterior average single-channel LZC as a function of dream experience class (only NE and DE awakenings), for the five participants (S0, S17, S19, S31, S36) that had both NE and DE awakenings from NREM2 sleep.

[a)] The gigantic (and meaningless) confidence intervals for the estimated marginal mean posterior LZC for S19, which had only one NE awakening and one DE awakening, suggest that this model should be disregarded.

**S0**	**Response**	**Fixed effect**	**df1**	**df2**	**F**	**sig.**			
	**LZC_{PHZ}**	*intercept*	1	1.6	843.840	.003			
		experience class	1	1.6	.369	.617		**sig. (pairwise comparison)**	
		**EMMs**	**estimate**	**std. err.**	**CI_{lower}**	**CI_{upper}**			*DE*
		NE	.466	.019	.373	.559			.617
		DE	.447	.025	.308	.586			
		**Repeated effect**		**estimate**	**std. err.**	**Z**	**sig.**	**CI_{lower}**	**CI_{upper}**
		AR1 diagonal (window)		.001	.001	1.24	.214	2.15e-4	.005
		AR1 rho (window)		.945	.044	21.29	<.001	.748	.989

**S17**	**Response**	**Fixed effect**	**df1**	**df2**	**F**	**sig.**			
	**LZC_{PHZ}**	*intercept*	1	7.5	6294.564	<.001			
		experience class	1	7.5	.084	.780		**sig. (pairwise comparison)**	
		**EMMs**	**estimate**	**std. err.**	**CI_{lower}**	**CI_{upper}**			*DE*
		NE	.465	.010	.442	.588			.780
		DE	.462	.006	.447	.476			
		**Repeated effect**		**estimate**	**std. err.**	**Z**	**sig.**	**CI_{lower}**	**CI_{upper}**
		AR1 diagonal (window)		3.31e-4	1.21e-4	2.74	.006	1.62e-4	6.76e-4
		AR1 rho (window)		.938	.023	41.43	<.001	.875	.970

**S19[a]**	**Response**	**Fixed effect**	**df1**	**df2**	**F**	**sig.**			
	**LZC_{PHZ}**	*intercept*	1	.25	1022.138	.306			
		experience class	1	.25	1.947	.661		**sig. (pairwise comparison)**	
		**EMMs**	**estimate**	**std. err.**	**CI_{lower}**	**CI_{upper}**			*DE*
		NE	.434	.020	-861.958	862.826			.661
		DE	.473	.020	-861.919	862.866			
		**Repeated effect**		**estimate**	**std. err.**	**Z**	**sig.**	**CI_{lower}**	**CI_{upper}**
		AR1 diagonal (window)		6.72e-4	.001	.498	.618	1.31e-5	.034
		AR1 rho (window)		.941	.119	7.889	<.001	-.285	.999

**S31**	**Response**	**Fixed effect**	**df1**	**df2**	**F**	**sig.**			
	**LZC_{PHZ}**	*intercept*	1	3.0	2233.346	< .001			
		experience class	1	3.0	1.734	.279		**sig. (pairwise comparison)**	
		**EMMs**	**estimate**	**std. err.**	**CI_{lower}**	**CI_{upper}**			*DE*
		NE	.469	.014	.423	.515			.279
		DE	.496	.014	.450	.542			
		**Repeated effect**		**estimate**	**std. err.**	**Z**	**sig.**	**CI_{lower}**	**CI_{upper}**
		AR1 diagonal (window)		6.86e-4	4.01e-4	1.71	.087	2.19e-4	.002
		AR1 rho (window)		.936	.037	25.01	<.001	.805	.980

**S36**	**Response**	**Fixed effect**	**df1**	**df2**	**F**	**sig.**			
	**LZC_{PHZ}**	*intercept*	1	6.7	5767.346	<.001			
		experience class	1	6.7	.538	.488		**sig. (pairwise comparison)**	
		**EMMs**	**estimate**	**std. err.**	**CI_{lower}**	**CI_{upper}**			*DE*
		NE	.491	.009	.469	.513			.488
		DE	.500	.009	.478	.522			
		**Repeated effect**		**estimate**	**std. err.**	**Z**	**sig.**	**CI_{lower}**	**CI_{upper}**
		AR1 diagonal (window)		5.58e-4	1.95e-4	2.86	.004	2.81e-4	.001
		AR1 rho (window)		.896	.037	24.19	<.001	.794	.948

References

Foulkes, W. D. (1962). Dream reports from different stages of sleep. *J. Abnorm. Soc. Psychol.* 65, 14–25.

Krol, L. R. (2020). EEG 10-10 system with additional information. Available at: https://commons.wikimedia.org/wiki/File:EEG_10-10_system_with_additional_information.svg [Accessed January 15, 2021].
